# The vacuolar morphology protein VAC14 plays an important role in sexual development in the filamentous ascomycete *Sordaria macrospora*

**DOI:** 10.1007/s00294-022-01244-0

**Published:** 2022-07-01

**Authors:** Anika Groth, Svenja Ahlmann, Antonia Werner, Stefanie Pöggeler

**Affiliations:** Department of Genetics of Eukaryotic Microorganisms, Institute of Microbiology and Genetics, Georg-August-University of Göttingen, Grisebachstr. 8, 37077 Göttingen, Germany

**Keywords:** VAC14, Fab1/PIKfyve-complex, Sexual development, Vacuolar morphology, *Sordaria macrospora*

## Abstract

The multiprotein Fab1p/PIKfyve-complex regulating the abundance of the phospholipid phosphatidylinositol 3,5-bisphosphate (PtdIns(3,5)P_2_) is highly conserved among eukaryotes. In yeast/mammals, it is composed of the phosphatidylinositol 3-phosphate 5-kinase Fab1p/PIKfyve, the PtdIns(3,5)P_2_ phosphatase Fig4p/Sac3 and the scaffolding subunit Vac14p/ArPIKfyve. The complex is located to vacuolar membranes in yeast and to endosomal membranes in mammals, where it controls the synthesis and turnover of PtdIns(3,5)P_2_. In this study, we analyzed the role and function of the Fab1p/PIKfyve-complex scaffold protein SmVAC14 in the filamentous ascomycete *Sordaria macrospora* (Sm). We generated the *Smvac14* deletion strain ∆vac14 and performed phenotypic analysis of the mutant. Furthermore, we conducted fluorescence microscopic localization studies of fluorescently labeled SmVAC14 with vacuolar and late endosomal marker proteins. Our results revealed that SmVAC14 is important for maintaining vacuolar size and appearance as well as proper sexual development in *S. macrospora*. In addition, SmVAC14 plays an important role in starvation stress response. Accordingly, our results propose that the turnover of PtdIns(3,5)P_2_ is of great significance for developmental processes in filamentous fungi.

**Supplementary Information:**

The online version contains supplementary material available at 10.1007/s00294-022-01244-0.

## Introduction

Conserved multiprotein kinase/phosphatase complexes tightly regulate developmental processes such as sexual development, cell fusion, cell migration and growth, as well as vesicular trafficking and organelle morphology. These include the Fab1p/PIKfyve-complex that mediates turnover and synthesis of phosphatidylinositol 3,5-bisphosphate (PtdIns(3,5)P_2_) at vacuolar membranes in yeast or on early- and late endosomes as well as multivesicular bodies (MVBs) and lysosomes in mammalian cells (Dove et al. 2002; Duex et al. 2006b; Ikonomov et al. 2009a; Jin et al. 2008; Rudge et al. 2004; Sbrissa et al. 2007; Shisheva 2008). The Fab1p/PIKfyve-complex is composed of the phosphatidylinositol 3-phosphate (PtdIns(3)P) 5-kinase Fab1p/PIKfyve, its antagonizing PtdIns(3,5)P_2_ phosphatase Fig4p/Sac3 and the scaffold protein Vac14p/ArPIKfyve (yeast/mammalian nomenclature) (Botelho et al. 2008; Duex et al. 2006a, b; Ikonomov et al. 2009b; Jin et al. 2008; Sbrissa et al. 2008; Schulze et al. 2014). In yeast, the complex additionally contains the Fab1p activator Vac7p and its inhibitor Atg18p (Bonangelino et al. 1997; Duex et al. 2006a, b; Efe et al. 2007; Gary et al. 2002). The low abundant phospholipid PtdIns(3,5)P_2_ controls diverse cellular functions including morphology of organelles, retrograde trafficking to the *trans*-Golgi network, ion transport, membrane recycling, cargo sorting into MVBs, acidification of endolysosomes and autophagy (de Lartigue et al. 2009; Dove et al. 2009; Efe et al. 2005; Rutherford et al. 2006; Shisheva 2008; Vicinanza et al. 2008). In mammals, disturbance of the abundance and distribution of the phospholipid PtdIns(3,5)P_2_ can cause severe developmental defects and neurodegeneration like Charcot–Marie–Tooth syndrome 4 J and amyotrophic lateral sclerosis (Chow et al. 2007, 2009; Zhang et al. 2007, 2008). In yeast, Vac14p forms a stable subcomplex with the 5-phosphatase Fig4p that allows for the recruitment of the Fab1p kinase (Duex et al. 2006b; Rudge et al. 2004). In this regard, both Vac14p and Fig4p were shown to activate Fab1p to regulate steady-state and hyperosmotic elevated levels of PtdIns(3,5)P_2_ (Bonangelino et al. 2002; Duex et al. 2006a, b; Gary et al. 2002). Mutants of Fab1p-complex components show low levels of PtdIns(3,5)P_2_ accompanied by enlarged less acidified vacuoles (Bonangelino et al. 2002; Duex et al. 2006a, b; Gary et al. 1998). Similar phenotypic effects were observed in mammalian cells either lacking or overexpressing Vac14 (Jin et al. 2008; Sbrissa et al. 2004; Schulze et al. 2014; Zhang et al. 2007). With a previously performed affinity approach, mammalian Vac14 was linked to proteins of the endosomal and autophagic pathways (Schulze et al. 2014).

Interestingly, the VAC14 homolog of the coprophilous ascomycete *Sordaria macrospora* (Sm) had been recently identified in a pulldown with the striatin-interacting phosphatase and kinase (STRIPAK)-complex component SCI1 (Reschka et al. 2018). The multiprotein STRIPAK-complex is conserved in animals and fungi. It coordinates a number of signaling pathways and developmental processes including cell-growth, -polarity and -migration, as well as vesicular trafficking, Golgi assembly, neural and sexual development, endocytosis, hyphal fusion, septation and vegetative growth (Beier et al. 2016; Bernhards and Pöggeler 2011; Bloemendal et al. 2012; Frey et al. 2015; Hwang and Pallas 2014; Kück et al. 2016, 2019; Pöggeler and Kück 2004; Shi et al. 2016). *S. macrospora* is used as model organism to study conserved processes like sexual development, meiosis and autophagy (Esser and Straub 1958; Kück et al. 2009; Pöggeler et al. 2006; Teichert et al. 2014, 2020). Both, autophagy and the SmSTRIPAK are important for proper fruiting-body formation and sexual development in *S. macrospora*. In this regard, we investigated the *S. macrospora* core scaffold protein SmVAC14 for the first time in a filamentous fungus. In this work, we generated and analyzed a *Smvac14* deletion mutant, ∆vac14 and performed localization studies using fluorescence microscopy. We showed that SmVAC14 is a conserved protein that partially co-localizes with vacuolar membranes and late endosomes. Moreover, *Smvac14* deletion caused enlarged vacuoles, deformed perithecia and impaired ascospore formation. Additionally, amino-acid starvation led to developmental defects in the ∆vac14 mutant though autophagy is apparently not affected.

## Materials and methods

### Strains, media and growth conditions

A list of all strains used and generated in this study is given in Table 1. For cloning and propagation of recombinant plasmids, *Escherichia coli* strain MACH1 (C862003, Thermo Fisher Scientific, Waltham, MA, USA) was used in standard culture conditions (Sambrook et al. 2001). To generate recombinant plasmids via homologous recombination (HR), positive transformants of the yeast *Saccharomyces cerevisiae* strain PJ69-4A were selected for uracil prototrophy (Colot et al. 2006; James et al. 1996). HR cloning in yeast bypasses traditional restriction digestion and ligation. The method relies on the generation of individually amplified PCR fragments with short 29 bp overlapping ends and co-transformation of these fragments with linearized yeast vectors for assembly by the yeast HR machinery (Colot et al. 2006). A *S. macrospora* Δku70 strain, defective in non-homologous end joining (Pöggeler and Kück 2006), was transformed with the recombinant plasmids according to the standard protocol (Kück and Hoff 2006; Walz and Kück 1995). *S. macrospora* is sensitive to the protein synthesis inhibitors hygromycin B and nourseothricin-dihydrogen sulfate. Using plasmids containing bacterial hygromycin B or nourseothricin resistance genes as dominant selection markers, positive transformants can be selected on media containing nourseothricin-dihydrogen sulfate (50 µg/mL, nat) (AB-102XL, Jena Bioscience GmbH, Jena, Germany) and/or hygromycin B (110 U/mL, hyg) (4,400,051-10MU, Merck, Kenilworth, NJ, USA) (Kück and Hoff 2006; Walz and Kück 1995). *S. macrospora* strains were grown on liquid or solid biomalt maize medium (BMM) or on solid Sordaria Westergaard (SWG) fructification medium under continuous light conditions at 27 °C (Elleuche and Pöggeler 2009; Esser 1982; Nowrousian et al. 2005). To generate single-spore isolates and strains expressing tagged proteins, *S. macrospora* strains were crossed as described previously (Bernhards and Pöggeler 2011).

**Table 1:** List of strains used and generated in this study

Strain	Genotype	Reference
***Escherichia coli***		
MACH1	Δ*recA1398*, *endA1*, *tonA*, *Φ80*Δ*lacM15*, Δ*lacX74*, *hsdR*, (rK-mK +)	Invitrogen
***Saccharomyces cerevisiae***		
PJ69-4A	*MATa*, *trp1-901*, *leu2-3*, *112*, *ura3-52*, *his3-200*, *gal4*Δ, *gal80*Δ, *LYS2::GAL1-HIS3*, *GAL2-ADE2*, *met2::GAL7-lacZ*	James et al. (1996)
***Sordaria macrospora***		
DSM997	wild type (wt)	DSMZ
S23442	mutation in *fus1-1* gene, brownish ascospores, fertile	Nowrousian et al. (2012)
∆ku70	∆ku70::*nat*^R^, fertile	Pöggeler and Kück (2006)
∆sci1	∆sci1::*hyg*.^R^, ssi, sterile	Reschka et al. (2018)
fus::RH2B^ect^	ectopic integration of pRH2B_hyg into S23442; *hyg*^R^, pt, fertile; *Pgpd::hh2b::tdTomato::TtrpC*	Reschka and Pöggler unpublished
wt::egfp^ect^	ectopic integration of p1783-1 into DSM997; *hyg*^R^, ssi, fertile; *Pgpd::egfp::TtrpC*	Voigt and Pöggeler (2013)
wt::TagRFP-T^ect^	ectopic integration of pTagRFP-T into DSM997; *nat*^R^, ssi, fertile; *Pccg1::TagRFP-T::TtrpC*	Werner et al. (2021)
wt::HA	ectopic integration of pHA_nat into DSM997; *nat*^R^, ssi, fertile; *Pccg1::HA::TtrpC*	Reschka et al. (2018)
wt::nbr1-egfp^ect^	ectopic integration of pnbr1-egfp into DSM997; *nat*^R^, ssi, fertile; *Pnbr1::nbr1::egfp::TtrpC*	Werner (2012)
wt::pom33-egfp^ect^	ectopic integration of p5’pom33-egfp into DSM997; *nat*^R^, ssi, fertile; *Ppom33::pom33::egfp::TtrpC*	Groth et al. (2021)
wt::egfp-atg8^ect^	ectopic integration of pegfp-atg8 into DSM997; *nat*^R^, ssi, fertile; *Patg8::egfp::atg8::Tatg8*	This study
wt::egfp-Ztrab5^ect^	ectopic integration of pHeGFPRab5_hyg into DSM997; *hyg*^R^, pt, fertile; *PZttub2::egfp::Ztrab5::TZttub2*	This study
wt::egfp-Ztrab7^ect^	ectopic integration of pHeGFPRab7_hyg into DSM997; *hyg*^R^, pt, fertile; *PZttub2::egfp::Ztrab7::TZttub2*	This study
∆vac14 (ssi 3.3, ssi 3.7, ssi 4.1)	∆vac14::*hyg*.^R^, ssi, fertile	This study
∆vac14::RH2B^ect^	ectopic integration of pRH2B_nat into ∆vac14; *hyg*^R^, *nat*^R^, pt, fertile; *Pgpd::hh2b::tdTomato::TtrpC*	This study
∆vac14::TagRFP-T^ect^	ectopic integration of pTagRFP-T into ∆vac14; *hyg*^R^, *nat*^R^, ssi, fertile; *Pccg1::TagRFP-T::TtrpC*	This study
∆vac14::5’vac14-TagRFP-T^ect^ (ssi 3.8 ssi 4.6, ssi 7.2)	ectopic integration of p5 ‘vac14-TagRFP-T into ∆vac14; *hyg*^R^, *nat*^R^, ssi, fertile; *Pvac14::vac14::TagRFP-T::TtrpC*	This study
∆vac14::ccg1vac14-TagRFP-T^ect^ (ssi 10.1.4, ssi 10.6, ssi 10.12.3)	ectopic integration of pccg1vac14-TagRFP-T_nat into ∆vac14; *hyg*^R^, *nat*^R^, ssi, fertile; *Pccg1::vac14::TagRFP-T::TtrpC*	This study
∆vac14::egfp-Ztrab5^ect^	ectopic integration of pegfp-Ztrab5_nat into ∆vac14; *hyg*^R^, *nat*^R^, pt, fertile; *Ptub2::egfp::Ztrab5::Ttub2*	This study
∆vac14::egfp-Ztrab7^ect^	ectopic integration of pegfp-Ztrab7_nat into ∆vac14; *hyg*^R^, *nat*^R^, pt, fertile; *Ptub2::egfp::Ztrab7::Ttub2*	This study
∆vac14::nbr1-egfp^ect^	ectopic integration of pnbr1-egfp into ∆vac14; *hyg*^R^, *nat*^R^, ssi, fertile; *Pnbr1::nbr1::egfp::TtrpC*	This study
∆vac14::egfp-atg8^ect^	ectopic integration of pegfp-atg8 into ∆vac14; *hyg*^R^, *nat*^R^, ssi, fertile; *Patg8::egfp::atg8::Tatg8*	This study
∆vac14::TagRFP-T-vac14^ect^	ectopic integration of pTagRFP-T-vac14 into ∆vac14; *hyg*^R^, *nat*^R^,pt**,** sterile; *Pvac14::TagRFP-T::vac14::Tvac14*	This study
wt::5’vac14- TagRFP-T^ect^	ectopic integration of p5 ‘vac14-TagRFP-T into DSM997; *nat*^R^, pt, fertile; *Pvac14::vac14::TagRFP-T::TtrpC*	This study
wt::ccg1vac14- TagRFP-T^ect^	ectopic integration of pccg1vac14-TagRFP-T_hyg into DSM997; *hyg*^R^, ssi, fertile; *Pccg1::vac14::TagRFP-T::TtrpC*	This study
wt::vac14-TagRFP-T + sci1-egfp^ect^	ectopic integration of pccg1vac14-TagRFP-T_hyg and p5’sci1-egfp into DSM997; *hyg*^R^, *nat*^R^, ssi, fertile; *Pccg1::vac14::TagRFP-T::TtrpC;* *Psci1::sci1::egfp::TtrpC*	This study
wt::vac14-TagRFP-T + egfp-vma1^ect^	ectopic integration of pccg1vac14-TagRFP-T_nat and pegfp-vma1 into DSM997; *hyg*^R^, *nat*^R^, pt, fertile; *Pccg1::vac14::TagRFP-T::TtrpC;* *Pvma1::egfp::vma1::Tvma1*	This study
wt::vac14-TagRFP-T + pom33-egfp^ect^	crossing of strain wt::ccg1vac14-TagRFP-T^ect^ with wt::pom33-egfp^ect^ *hyg*^R^, *nat*^R^, ssi, fertile; *Pccg1::vac14::TagRFP-T::TtrpC;* *Ppom33::pom33::egfp::TtrpC*	This study
wt::vac14-TagRFP-T + egfp-Ztrab5^ect^	ectopic integration of pccg1vac14-TagRFP-T_hyg and pegfp-Ztrab5_nat into DSM997; *hyg*^R^, *nat*^R^, ssi, fertile; *Pccg1::vac14::TagRFP-T::TtrpC;* *Ptub2::egfp::rab5::Ttub2*	This study
wt::vac14-TagRFP-T + egfp-Ztrab7^ect^	ectopic integration of pccg1vac14-TagRFP-T_hyg and pegfp-Ztrab7_nat into DSM997; *hyg*^R^, *nat*^R^, ssi, fertile; *Pccg1::vac14::TagRFP-T::TtrpC;* *Ptub2::egfp::rab7::Ttub2*	This study
wt::vac14-TagRFP-T + nbr1-egfp^ect^	crossing of strain wt::ccg1vac14-TagRFP-T^ect^ with wt::nbr1-egfp^ect^ *hyg*^R^, *nat*^R^, ssi, fertile; *Pccg1::vac14::TagRFP-T::TtrpC;* *Pnbr1::nbr1::egfp::TtrpC*	This study
wt::vac14-TagRFP-T + egfp-atg8^ect^	crossing of strain wt::ccg1vac14-TagRFP-T^ect^ with wt::egfp-atg8^ect^ *hyg*^R^, *nat*^R^, ssi, fertile; *Pccg1::vac14::TagRFP-T::TtrpC;* *Patg8::egfp::atg8::Tatg8*	This study

*nat*^*R*^ nourseothricin resistant, *hyg*^*R*^ hygromycin resistant, *ssi* single-spore isolate, *pt* primary transformant, *ect* ectopically integrated, *P* promoter, *T* terminator, *Pgpd* promoter of the glyceraldehyde-3-phosphate dehydrogenase gene from *Aspergillus* *nidulans*, *Pccg1* promoter of the *clock-controlled gene 1* from *Neurospora* *crassa*, *TtrpC* terminator of the anthranilate synthase gene from *A.* *nidulans*, *egfp*: gene for enhanced green fluorescent protein (EGFP) from *Aequorea* *victoria*, *TagRFP-T* gene for red fluorescence protein TagRFP-T of *Entacmaea quadricolor*, *tdTomato* gene for red fluorescence protein tdTomato from *Discosoma* species

### Phenotypic analysis

For phenotypic analysis, three biological replicates each of the *S. macrospora* wt, ∆vac14, ∆vac14::5’vac14-TagRFP-T^ect^ and ∆vac14::ccg1vac14-TagRFP-T^ect^ strains were grown on solid SWG medium at 27 °C under continuous light conditions. Strains were documented with a VHX-550F Digital Microscope (Keyence, Neu-Isenburg, Germany). For quantification of perithecia, strains were grown for 7 days, and perithecia present per 0.0625 cm^2^ were counted. This was done 20 times and the whole experiment was repeated three times. For phenotypic analysis of perithecia, cross-sections of agar plates were prepared. To assess ascus rosette maturation, an “inner” and “outer” area of the petri dish was defined using the diameter (2.8 cm) of a 50 mL falcon tube as spacer. For these analyses, the strains were grown for 8 days. Perithecia were prepared using dissecting needles and thin agar slices were prepared with a scalpel and both were placed on a glass slide for documentation (Werner et al. 2019). For determination of ascospore maturation, strains were grown for 9 days, ten perithecia per strain were cracked and the enclosed ascus rosettes were categorized into four categories: (a) rosettes containing predominantly asci with 8 mature spores, (b) rosettes containing frequently asci with 8 black spores, (c) rosettes containing predominantly asci with immature spores, (d) rosettes containing only asci with immature spores. The ascospore length and width was determined for 2 complete asci (in total 16 spores) for three biological replicates of each strain (in total 48 spores for each strain). To determine the vegetative growth rate and sexual development under different stress conditions, strains were grown over 10 days on SWG media supplemented with 0.1 M NaCl, 0.4 M sorbitol, 2.5 mM 3-amino-1,2,4-triazole (3-AT), 0.003% SDS, 0.01% H_2_O_2_ or without KNO_3_. This experiment was repeated two times.

The determination of the growth rate/day was done in triplicate and strains were grown in 30-cm race tubes filled with the respective stress media. After 3 days of growth, the growth front was marked every day at the same time. This experiment was repeated three times. The experiment for analyzing sexual development was repeated two times.

### Construction of plasmids

All plasmids used and constructed in this study are shown in Tab. S1. Plasmids were generated via HR in *S. cerevisiae* (Colot et al. 2006), or Golden Gate (GG) cloning (Dahlmann et al. 2021). Information about the used primers (Sigma-Aldrich Chemie GmbH Taufkirchen, Germany) is provided in Tab. S2. For the generation of the pvac14-KO_V3w knockout plasmid, we amplified the first 1030 bp (5’-flanking region) and the last 1030 bp (3’-flanking region) of the *vac14* open reading frame (ORF) from *S.* *macrospora* wt genomic (g)DNA. For the 5’ flanking region, we used primer pair Vac14-ko-5f_3w/Vac14-ko-5r_3w and for the 3’ flanking region primer pair Vac14-ko-3f_3/Vac14-ko-3r_3. The resulting two PCR fragments together with the donor vector pGG-hph and the destination vector pDest-Amp were mixed in a 2:2:2:1 ratio and the GG reaction was performed in a PCR cycler as described by (Dahlmann et al. 2021).

For the construction of p5’vac14-TagRFP-T, a fragment of 4307 bp containing the *S.* *macrospora Pvac14* and *vac14* ORF was amplified from wt gDNA using the primer combination Vac14-egfp-f/Vac14-tRFP-r. Together with a fragment (1531 bp) comprised of *TagRFP-T* and the *TtrpC* terminator of *A.* *nidulans* amplified from pTagRFP-T (Werner et al. 2021) with the primers RFP-f and pRS426GFPrev, both fragments were integrated into *Xho*I-linearized pRS-nat (Klix et al. 2010) via HR in the *S. cerevisiae* strain PJ69-4A (Colot et al. 2006).

To construct the overexpression plasmids pccg1vac14-TagRFP-T_nat/_hyg, following three fragments were cloned into *Xho*I-linearized pRS-nat (Klix et al. 2010) or pRS-hyg (Bloemendal et al. 2012), respectively. The overexpression promoter of the *clock-controlled gene 1* (*Pccg1*) of *Neurospora* *crassa* (950 bp) was amplified with the primer combination pRSccg1/Pccg1-r from pHA_nat (Reschka et al. 2018). The *S. macrospora vac14* ORF (3439 bp) was amplified from wt gDNA using the primer pair Vac14-ccg1-f/Vac14-tRFP-r and a fragment (1513 bp) consisting of *TagRFP-T* and the *TtrpC* of *A.* *nidulans* was amplified from pTagRFP-T (Werner et al. 2021) with the primers RFP-f and pRS426GFPrev. Fusion of the three PCR products was performed via HR in *S. cerevisiae* (Colot et al. 2006). For tagging SmVAC14 N-terminally with TagRFP-T, plasmid pTagRFP-T-vac14 was generated with the NEBuilder HiFi DNA Assembly Cloning Kit (New England Biolabs, Ipswich, MA, USA) according to the instruction manual. The promoter *Pvac14* (882 bp) was amplified from wt gDNA using the primer combination N-vac14_P-f/N-vac14_P-r, the *TagRFP-T* (746 bp) was amplified from pTagRFP-T (Werner et al. 2021) with the primers N-tRFP-f and N-tRFP-r, and a fragment (4201 bp) consisting of the *vac14* ORF and terminator (*Tvac14*) was amplified from wt gDNA with primer combination N-vac14-f/N-vac14_T-r. The three fragments were cloned into *EcoR*V-linearized pJet_nat (Nordzieke, unpublished).

For the generation of the plasmids pegfp-Ztrab5/-Ztrab7_nat/hyg, primer pair Tub2Ztf/Tub2Ztr was used to amplify a fragment consisting of the constitutive *tub2* promoter of *Zymoseptoria tritici* (Zt) (*PZttub2*), *egfp*, the *Ztrab5/Ztrab7* coding region and the constitutive *Zttub2* terminator (*TZttub2*) from the plasmids pHeGFPRab5/-Rab7_hyg (Kilaru et al. 2015). The resulting fragments of 3763 bp and 3766 bp were integrated into *Xho*I-linearized pRS-nat (Klix et al. 2010) or pRS-hyg (Bloemendal et al. 2012), respectively, via HR in the *S. cerevisiae* strain PJ69-4A (Colot et al. 2006).

The plasmid pegfp-vma1, was constructed by amplifying the *S. macrospora vma1* native promoter (1058 bp) and ORF including the *vma1* terminator (4068 bp) with primer pairs Vma1P-f/Vma1P-EGFP-r and Vma1-EGFP-f/Vma1-r, respectively, from wt gDNA. The *egfp* fragment (717 bp) was amplified with the primers GFP-f and GFP-r from p1783-1 (Pöggeler et al. 2003) and the fragments were cloned into *Xho*I-linearized pRS-hyg via HR in *S. cerevisiae* (Colot et al. 2006).

Sequencing of generated plasmid DNA was performed by Seqlab Sequence Service Laboratories GmbH (Göttingen, Germany).

### Generation of the *S. macrospora* knockout strain ∆vac14

For the partial deletion of the *S. macrospora vac14* gene (Fig. S1), the pvac14-KO_V3w knockout plasmid was used as template to amplify the 3526 bp deletion cassette with the primer pair GG_KO_fw/GG_KO_rv, containing the defined 5’- and 3’-flanking regions of *vac14* and the *hph* cassette. The *S. macrospora* ∆ku70 strain (Pöggeler and Kück 2006) was transformed with the deletion cassette to replace the remaining 1379 bp of the *vac14* ORF with the *hph* cassette (Fig. S1a). Crosses of primary transformants with the color-spore mutant fus1-1 were performed as described previously (Bernhards and Pöggeler 2011; Nowrousian et al. 2012). Single-spore isolates of three independent ∆vac14 mutants carrying *hyg* resistance were selected and verification of the absence of the fragment of the *vac14* gene and integration of the *hph* cassette at the desired locus was performed with primer pairs Vac14-2v5f/ Vac14-2vORF5-r (3395 bp) and tC1_o/Vac14-2v3r (2555 bp), respectively (Fig. S1b). To verify the presence of the *ku70* gene in ∆vac14 after crossing, primer pair Smku70-v1-f/ku70-ko-v3f(R) (2851 bp) was used (Fig. S1b). For Southern hybridization, gDNA of the *S.* *macrospora* wt, ∆ku70 and ∆vac14 strain was digested with *Pst*I. A capillary blot using a nylon membrane (RPN303B, GE Healthcare, Boston, MA, USA) was performed overnight at RT. The 1030 bp 3’-probe was amplified from *S.* *macrospora* wt gDNA with the Vac14-ko-3f_3/Vac14-ko-3r_3 primer pair. Labeling of the probe was performed with the Amersham AlkPhos Direct Labelling and Detection Kit (RPN3680, GE Healthcare, Botson, MA, USA). Detection was done according to the manufacturer’s manual. Signals were visualized on X-ray films (Amersham Hyperfilm™ ECL, GE Healthcare, Botson, MA, USA) using an “Optimax X-ray film processor” (PROTEC GmbH & Co. KG, Oberstenfeld, Germany) (Fig. S1c and Fig. S2a).

### Light and fluorescence microscopy

To investigate vegetative hyphae and sexual structures, *S. macrospora* strains were grown on SWG-covered glass slides for 5 days or on solid SWG medium for 9 days under continuous light at 27 °C. The slides were prepared as described previously (Groth et al. 2021), whereas SWG was used as solid medium and instead of liquid BMM water was poured into the petri dish to prevent desiccation of the growth medium. The documentation was performed with an AxioImage M1 microscope (Zeiss, Jena, Germany) using differential interference contrast (DIC) or a VHX-500F Digital Microscope (Keyence, Neu Isenburg, Germany). Images were captured with a Photometrix CoolSNAP HQ camera (Roper Scientific, Photometrics, Tuscon, AZ, USA). Image processing was done using ZEISS ZEN Digital Imaging (version 2.3; Zeiss, Jena, Germany) and the Affinity Publisher software (version 1.10.1, Serif (Europe) Ltd., Nottingham, UK, https://affinity.serif.com/de/publisher/; accessed on 24.08.2021).

For fluorescence microscopic analyses, *S. macrospora* strains were grown for 24 h on BMM-agar slides, as described in Groth et al. (2021), for 72 h on solid SWG medium supplemented with 1.5% agarose (Biozym Scientific GmbH, Hessisch Oldendorf, Germany), or for 24–72 h on SWG + 1.5% agarose media supplemented with 0.1 M NaCl, 0.4 M sorbitol, 2.5 mM 3-AT, 0.003% SDS, 0.01% H_2_O_2_ or without KNO_3_ at 27 °C under continuous light conditions. To detect EGFP signals, Chroma filter set 49,002 (exciter ET470/40x, ET525/50 m, beamsplitter T495lpxr), for TagRFP-T/tdTomato/FM4-64-signals, Chroma filter set 49,005 (exciter ET545/30x, emitter ET620/60 m and beamsplitter T570LP) and for CMAC, Chroma filter set 49,000 (ET350/50x, emitter ET460/50 m and beamsplitter T400LP) was used.

For FM4-64 (Thermo Fisher Scientific, Waltham, MA, USA) staining, *S. macrospora* strains were grown on solid SWG + 1.5% agarose for 24 h at 27 °C. Staining was conducted by applying 100 µL of an FM4-64 solution (1 µg/mL in distilled water) to the mycelium on the agar piece followed by incubation for 15 min at 37 °C.

For CMAC (Thermo Fisher Scientific, Waltham, MA, USA) staining, *S. macrospora* strains were grown on BMM-slides or over a piece of cellophane (0.5 cm × 0.5 cm) on solid SWG for 24 h or on solid SWG + 1.5% agarose for 72 h at 27 °C. Then, the CMAC 10 mM stock solution was diluted 1:400 in distilled water and 100 µL of the CMAC solution was applied for 30 min at 37 °C to the mycelium.

With the transformation of plasmid pRH2B_nat (histone 2B fused with tdTomato) (Reschka et al. 2018) into the ∆vac14 deletion strain, nuclei were visualized by fluorescence microscopy.

For time lapse studies of growing hyphae, *S. macrospora* strains were grown on BMM + 1.5% agarose for 24 h at 27 °C, as described previously (Groth et al. 2021). Recording intervals of 5 s over 20 min were used for time lapse studies.

### Protein sample preparation and Western blot hybridization

For protein extraction from fungal mycelium, *S. macrospora* strains were cultivated in liquid BMM and were grown for 3 days at 27 °C. Then, the mycelium was harvested, dried, ground in liquid nitrogen and 520 µL of lysis buffer (10 mM Tris–HCl pH 7.5, 150 mM NaCl, 0.5 mM EDTA pH 8.0, 1 mM PMSF, 2 mM DTT, 0.5% NP-40, 1 × protease inhibitor cocktail IV (1tbl/50 mL, 04,693,132,001, Mannheim, Germany), 1 × PhosSTOP™ (1tbl/10 mL, 04,906,837,001, Roche, Mannheim, Germany)) per g mycelium powder was added.

Cells were lysed in a Tissue Lyser (Qiagen, Hilden, Germany) by 30 Hrz for 2 min and prepared for Western Blot analysis by applying 4 × NuPAGE® LDS-SB (NP0007, Thermo Fisher Scientific, Waltham, MA, USA) according to the manufacturer’s manual. As protein standards, either the Nippon Genetics Co. Europe blue star pre-stained protein marker (MWP03, NIPPON Genetics Europe, Düren, Germany) or the PageRuler™ pre-stained protein ladder (26,619, Thermo Fisher Scientific, Waltham, MA, USA) were used.

Proteins were separated by SDS-PAGE and transferred to a Amersham™ Protran™ Nitrocellulose Blotting Membrane (RPN203B, GE Healthcare, Little Chalfont, UK) using 1 × transfer buffer and a Mini Trans-Blot® Cell device as described by the manufacturer (Bio-Rad Laboratories, Hercules, CA, USA) (Towbin et al. 1979).

The nitrocellulose membrane, containing transferred proteins, was blocked with 5% (w/v) skim milk powder in 1 × Tris-buffered saline supplemented with 0.05% Tween 20® (TBST) for 1 h at RT. Detection of antigen–antibody reaction was performed with a primary EGFP (rat)- (1:4000, 3h9-100, ChromoTek GmbH, Planegg-Martinsried, Germany) or TagRFP-T (rabbit) -antibody (1:12,500, AB233-ev, BioCat (Evrogen, Moscow, Russia)) solved in 5% skim milk/TBST. The membrane and antibody solution were incubated overnight at 4 °C. After the primary antibody was removed, the membrane was washed three times with 1 × TBST for 15 min. A horse-radish peroxidase (HRP) coupled secondary anti rat- or rabbit-antibody (1:5500, 62–9520, Thermo Fisher Scientific, Waltham, MA, USA; 1:5000, G-21234, Thermo Fisher Scientific, Waltham, MA, USA) was applied to the membrane for 1 h at RT before the membrane was washed three times with 1 × TBST for 15 min. Enhanced chemiluminescence reaction was used to detect the HRP-coupled antibodies using the Immobilon™ Western HRP Substrate kit (WBKLS0500, Merck, Kenilworth, NJ, USA). Signals were visualized on X-ray films (Amersham Hyperfilm™ ECL, GE Healthcare, Botson, MA, USA) using an “Optimax X-ray film processor” (PROTEC GmbH & Co. KG, Germany).

### Protein domain determination

Protein domains were predicted using the program InterProScan (https://www.ebi.ac.uk/interpro/search/sequence/; accessed on 15.03.2021) (Blum et al. 2021). The coiled-coil motifs were predicted using NPS@: COILED-COILS PREDICTION (https://npsa-prabi.ibcp.fr/cgi-bin/npsa_automat.pl?page=/NPSA/npsa_lupas.html; accessed on 15.03.2021) (Lupas et al. 1991). Transmembrane domains (TMD) were predicted with the program HMMTOP (http://www.enzim.hu/hmmtop/html/submit.html; accessed on 15.03.2021) (Tusnády and Simon 2001). Design of the schematic illustration was performed in same relation to the amino acids indicated in the figure using the Affinity Publisher software (version 1.10.1, Serif (Europe) Ltd., Nottingham, UK, https://affinity.serif.com/de/publisher/; accessed on 24.08.2021).

### Multiple sequence alignment and phylogenetic analysis of VAC14

Protein sequences of VAC14 from fungi, animals and plants were obtained from BLASTP search using the public databases at NCBI (https://blast.ncbi.nlm.nih.gov/Blast.cgi?PAGE=Proteins; accessed on 24.08.2021) and were prepared with the online program MAFFT (version 7, https://mafft.cbrc.jp/alignment/server/; accessed on 24.08.2021) (Katoh et al. 2019). The program GeneDoc (version 2.7.000; accessed on 24.08.2021) (Nicholas and Nicholas 1997) and the Affinity Publisher software (version 1.10.1, Serif (Europe) Ltd., Nottingham, UK, https://affinity.serif.com/de/publisher/; accessed on 24.08.2021) were used to represent the alignment of protein sequences. Alignments of multiple protein sequences and neighbor joining phylogenetic analysis were performed with MAFFT (version 7, https://mafft.cbrc.jp/alignment/server/; accessed on 12.10.2021) (Katoh et al. 2019). To test the tree for statistical significance, a bootstrap analysis was conducted with 1000 iterations. The tree was displayed with Phylo.io (version 1.0.k, http://phylo.io/; accessed on 12.10.21) (Robinson et al. 2016) and edited with the Affinity Publisher software (version 1.10.1, Serif (Europe) Ltd., Nottingham, UK, https://affinity.serif.com/de/publisher/; accessed on 12.10.2021).

## Results

### The VAC14 protein is conserved among fungi, plants and animals

**Fig. 1:**
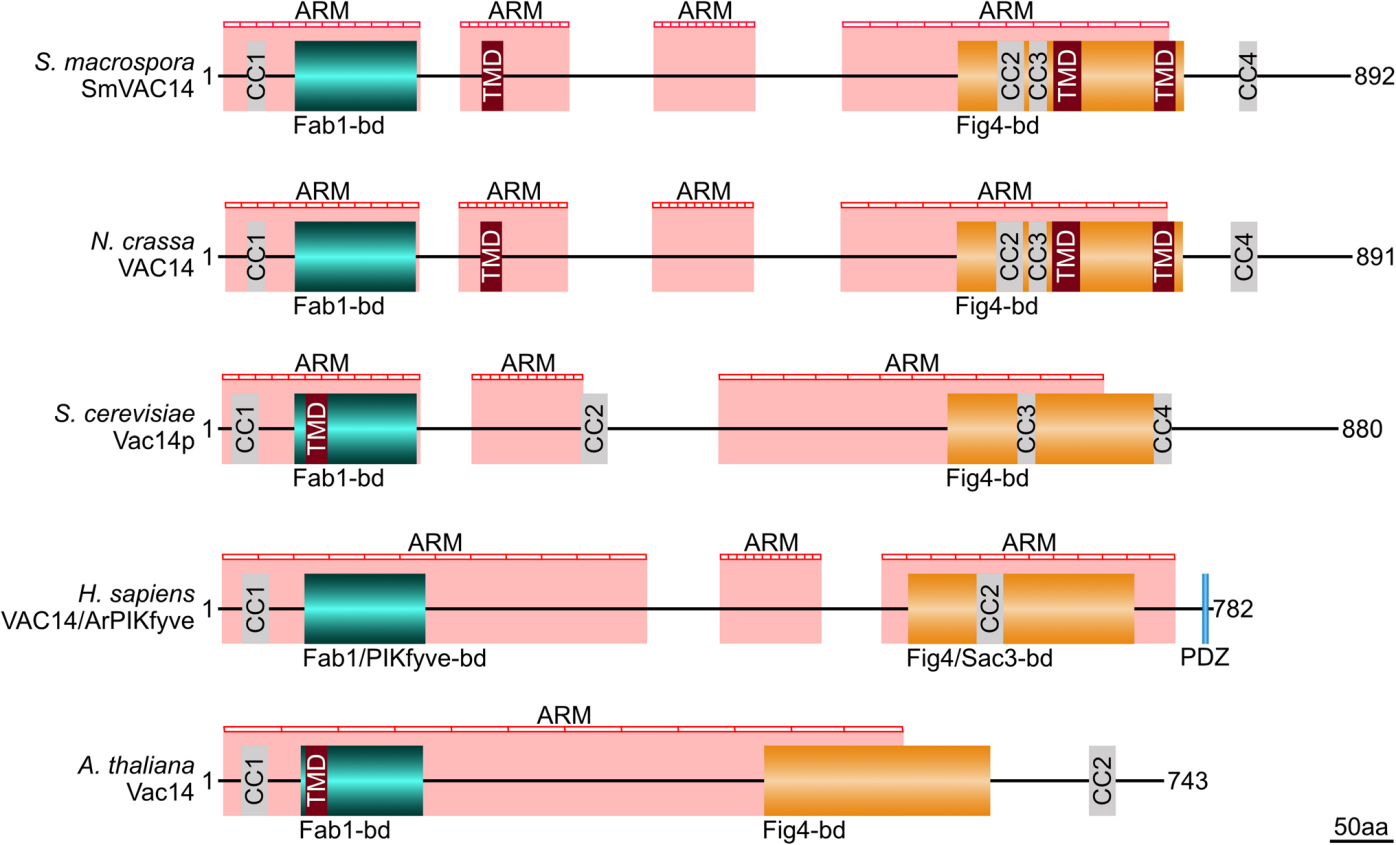
Domain organization of VAC14 proteins from fungi, animals and plants. Domains for Fab1 and Fig4 binding and ARM-repeats were predicted using the program InterProScan (Blum et al. 2021). Coiled-coil (CC) motifs (light gray) were predicted with NPS@: COILED-COILS PREDICTION (Lupas et al. 1991) and transmembrane domains (TMD) (dark red) with HMMTOP (Tusnády and Simon 2001). The N-terminal Fab1-binding domain (Fab1-bd) is shown in cyan, the C-terminal Fig4bd in orange, and a PSD95-Discs-large-ZO-1 (PDZ)-recognition motif (Lemaire and McPherson 2006) in light blue. Positions of presumable Armadillo (ARM)-repeats are indicated as striped red bars. Protein sequence of the *S. macrospora* SmVAC14 (SMAC_08299) was taken from the *S. macrospora*-specific peptide database Smacrospora_v03 (Blank-Landeshammer et al. 2019). Accession numbers of the other proteins are as following: *N. crassa* VAC14 (XP_011395167.1),* S. cerevisiae* Vac14p (NP_013490.3),* H. sapiens* VAC14/ArPIKfyve (NP_060522.3) and *A. thaliana* Vac14 (NP_565275.1)

In previously performed LC–MS analysis with the SmSTRIPAK-complex component SCI1 as bait, a protein encoded by SMAC_08299 had been identified and predicted to be a VAC14 homolog via BLASTP analysis (Reschka et al. 2018). Moreover, interactors of the endolysosomal and autophagic pathways were identified for mammalian VAC14 (Schulze et al. 2014). Since the SmSTRIPAK-complex and the autophagic process are critical for *S. macrospora* sexual development, we investigated the SmVAC14 protein in more detail (Voigt and Pöggeler 2013; Werner et al. 2019). The 3384-bp coding region of the *S. macrospora vac14* gene is interrupted by 8 introns and encodes a protein of 892 aa with a molecular weight of 98 kDa (from the genome database Smacrospora_v03 from (Blank-Landeshammer et al. 2019). SmVAC14 is predicted to contain a Fab1- and a Fig4-binding domain, 3 transmembrane domains (TMD), 4 Coiled Coils (CC), and with up to 4 predicted Armadillo (ARM)-repeats it belongs to the ARM-repeat superfamily. The ARM-superfamily also contains HEAT (huntingtin-elongation-A subunit-TOR)-repeats but due to their degeneration and prediction problems of commonly used software (Andrade et al. 2001), we focused on the prediction of ARM-repeats. Domain organization of VAC14 proteins in *S. macrospora*, *N.* *crassa*, *Saccharomyces cerevisiae*, *Homo sapiens* and *Arabidopsis thaliana* are shown in Fig. 1. Multiple sequence alignment with the SmVAC14 protein sequences using the online tool MAFFT (Katoh et al. 2019,2019) revealed 97% sequence similarity with the *N. crassa* NcVAC14 protein (XP_011395167.1), 52% with the *S. cerevisiae* Vac14p (NP_013490.3), 47% with the *H. sapiens* HsVAC14/ArPIKfyve protein (NP_060522.3) and 50% with the *A. thaliana* AtVac14 protein (NP_565275.1), respectively (Fig. S3). Furthermore, multiple sequence alignment revealed that VAC14 is conserved in saprophytic and pathogenic species among the clades of Ascomycota and Basidiomycota showing 55–86% sequence similarity to SmVAC14 (Fig. S4 and Fig. S5).

### Deletion of *vac14* results in deformed perithecia and an impairment of ascospore formation

**Fig. 2:**
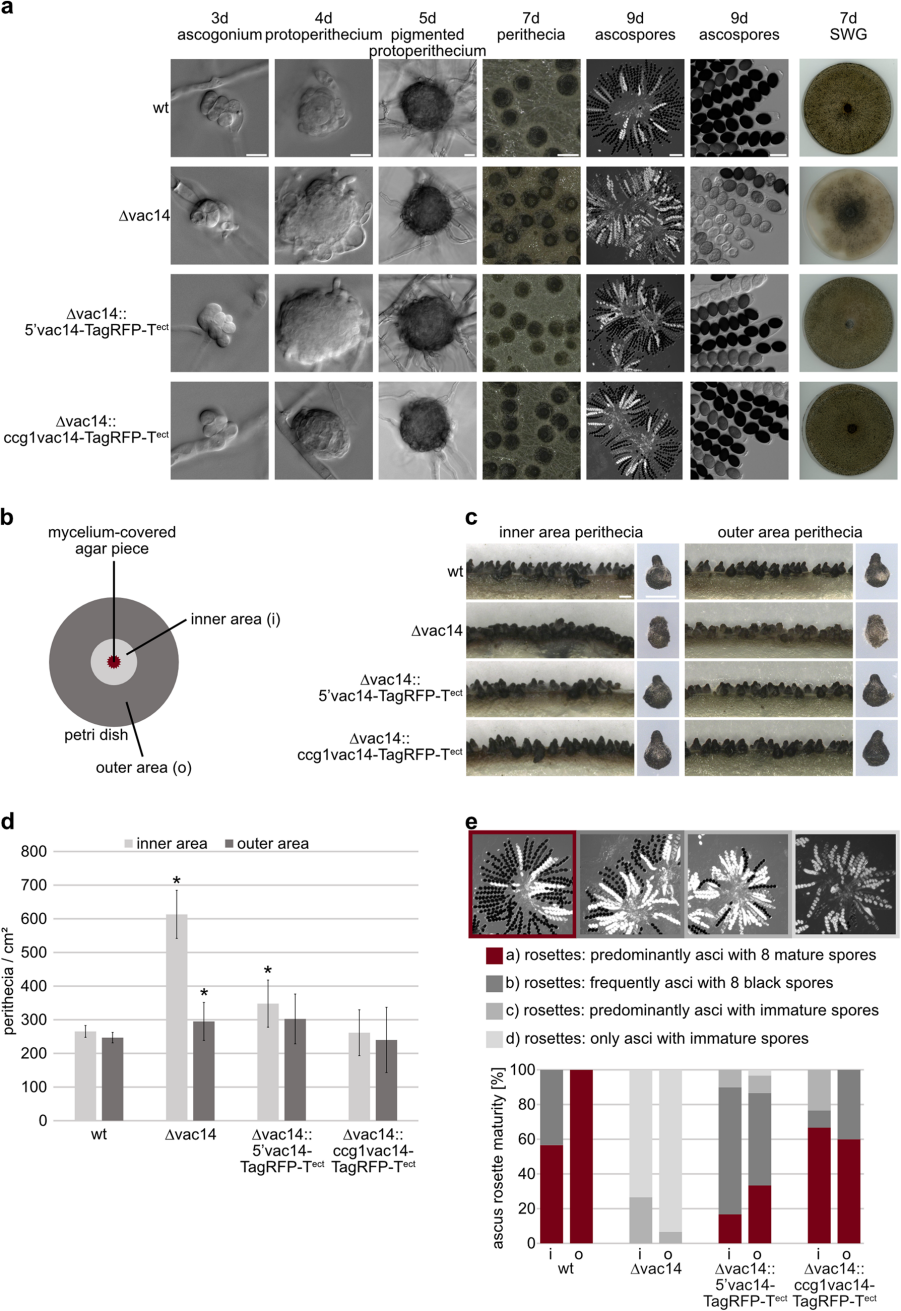
Phenotypic analysis of the *S.* *macrospora* wt, ∆vac14 and complementation strains ∆vac14::5’vac14-TagRFP-T^ect^ and ∆vac14::ccg1vac14-TagRFP-T^ect^. **a** Microscopic investigation of sexual development. Strains were grown on SWG slides or on solid SWG medium at 27 °C for indicated periods of 3–9 days. Scale bars from left to right: 10 µm; 10 µm; 10 µm; 0.5 mm; 100 Mm and 25 µm. **b** Schematic illustration of a petri dish divided in an inner (i) (dark gray) and outer (o) (light gray) area with the inoculum, mycelium-covered agar piece (dark red), placed upside-down in the center. **c** Cross sections of the strains from the defined inner and outer area and representative pictures of a single perithecium. Pictures were taken after strains were grown for 8 days on solid SWG media. Scale bar: 0.5 mm. **d** Quantification of perithecia per cm^2^ after 7 days of growth. Perithecia were counted 20 times in an area of 0.0625 cm^2^ and the averages from three biological replicates from each strain of three independent experiments (*n* = 60) are shown. Counting was performed in the inner (i) (dark gray) and outer (o) (light gray) area, respectively. Significant differences to the wt of *p* < 0.05 according to Student’s t-test are indicated by asterisks (*). **e** Ascus rosette maturation was determined after 9 days on solid SWG media. Ten perithecia of three biological replicates from each strain (*n* = 30) in the defined inner (i) and outer (o) area were cracked and categorized into four categories: (a) rosettes: predominantly asci with 8 mature spores (dark red), (b) rosettes: frequently asci with 8 black spores (dark gray), (c) rosettes: predominantly asci with immature spores (middle gray), (d) rosettes: only asci with immature spores (light gray). A representative picture of an ascus rosette of each category is shown above the diagram. Mature black spores of wt in the outer area were set to 100%

The *S. macrospora* ∆vac14 partial-deletion mutant was generated using the ∆ku70 strain (Pöggeler and Kück 2006). For the construction of the ∆vac14 strain, homologous recombination of a *hph* deletion cassette flanked by the first and last 1000 bp of the *vac14* gene was performed resulting in a 1140-bp deletion of the *vac14* coding region. Partial deletion of *vac14* was confirmed by PCR and Southern blot analysis for three single-spore isolates (Fig. S1 and Fig. S2). To investigate the role of SmVAC14 during sexual development, the life cycle of the ∆vac14 deletion strain (exemplarily shown for ssi 3.3) and two complementation strains were microscopically examined and compared to the wt (Fig. 2a). In the complementation strains, VAC14 is C-terminally tagged with TagRFP-T either under the control of the endogenous promotor (5’) (∆vac14::5’vac14-TagRFP-T^ect^) or the overexpression promotor (*ccg1*) (∆vac14::ccg1vac14-TagRFP-T^ect^). Further, phenotypic analyses were performed in which the morphology and number of perithecia and ascus rosette maturity was determined in the strains (Fig. 2b–e and Fig. S2b).

All strains completed the life cycle within 9 days including the production of ascospores. The *S. macrospora* life cycle begins with a germinating ascospore that develops into a vegetative mycelium. After 2–3 days, ascogonia, the female gametangia, were formed and after 3–4 days protoperithecia, unpigmented fruiting-body precursors, were produced. These stages were observed in all strains, however the ∆vac14 mutant and the vac14 complementation strain with its own promoter displayed enlarged protoperithecia (Fig. 2a). The vac14 complementation strain with the overexpression promoter gave rise to normal sized protoperithecia. The less effective complementation with the endogenous promoter might be due to the ectopic integration of complementation constructs. While the integration locus might influence the expression of *vac14* under control of its endogenous promoter, overexpression of *vac14* at an ectopic locus might overcome this insufficient expression.

After further development of the protoperithecia into melanin-pigmented protoperithecia, self-fertilization, karyogamy, meiosis and a postmeiotic-mitosis in the maturing perithecia took place. Subsequently, eight linear-arranged black ascospores are present per ascus. In ∆vac14, the ascospores are predominantly immature compared to the wt. After growth for 7 days on SWG medium, the ∆vac14 deletion mutant exhibited an increased density of perithecia formed near the agar piece in the center of the petri dish (Fig. 2a). Due to this phenotype, we defined an “inner” and “outer” area of the petri dish for further analysis (Fig. 2b). Cross sections were performed to analyze the morphology of the perithecia in the inner and outer area. This showed that the perithecia of the inner area of the ∆vac14 strain appeared more melanized and piled up. In addition, they appeared deformed and do not form a neck and a pear-shaped structure as seen in the wt or complementation strains (Fig. 2c). To investigate the ∆vac14 phenotype regarding perithecia production in more detail, numbers of perithecia per cm^2^ in the defined areas were calculated after 7 days (Fig. 2d). This analysis revealed a significantly higher number (~ twofold) of perithecia in the inner area of the ∆vac14 strain compared to the wt. For further phenotypic analysis, ascus rosette maturation was analyzed in the defined areas in all strains (Fig. 2e). For this, ascus rosettes were categorized into 4 categories according to their maturity revealing that ∆vac14 perithecia contain an increased number of asci with immature spores, as well as asci with less than eight ascospores, when compared to the wt. This defect was only partially reverted in the complementation strains, whereby the vac14 complementation strain with the overexpression promoter produced more mature asci than the complementation strain with the endogenous promoter. It also appeared that the form and size of ascospores of Δvac14 strain differed from wt ascospores. Ascospores of Δvac14 are more round than in wt and complementation strains (Fig. 2a). Therefore, we measured the length and width of ascospores from wt, Δvac14 and the complementation strains. The length of ascospores from the Δvac14 mutant and the two complementation strains was significantly decreased in comparison to wt ascospores. However, only the width of ascospores from Δvac14 increased significantly (Fig. S6). As a consequence, the ratio of ascospore length and width decreased significantly in the Δvac14 mutant strain. This defect was fully reverted in the complementation strains (Fig. S6).

### Vacuolar morphology is altered in the ∆vac14 mutant

**Fig. 3:**
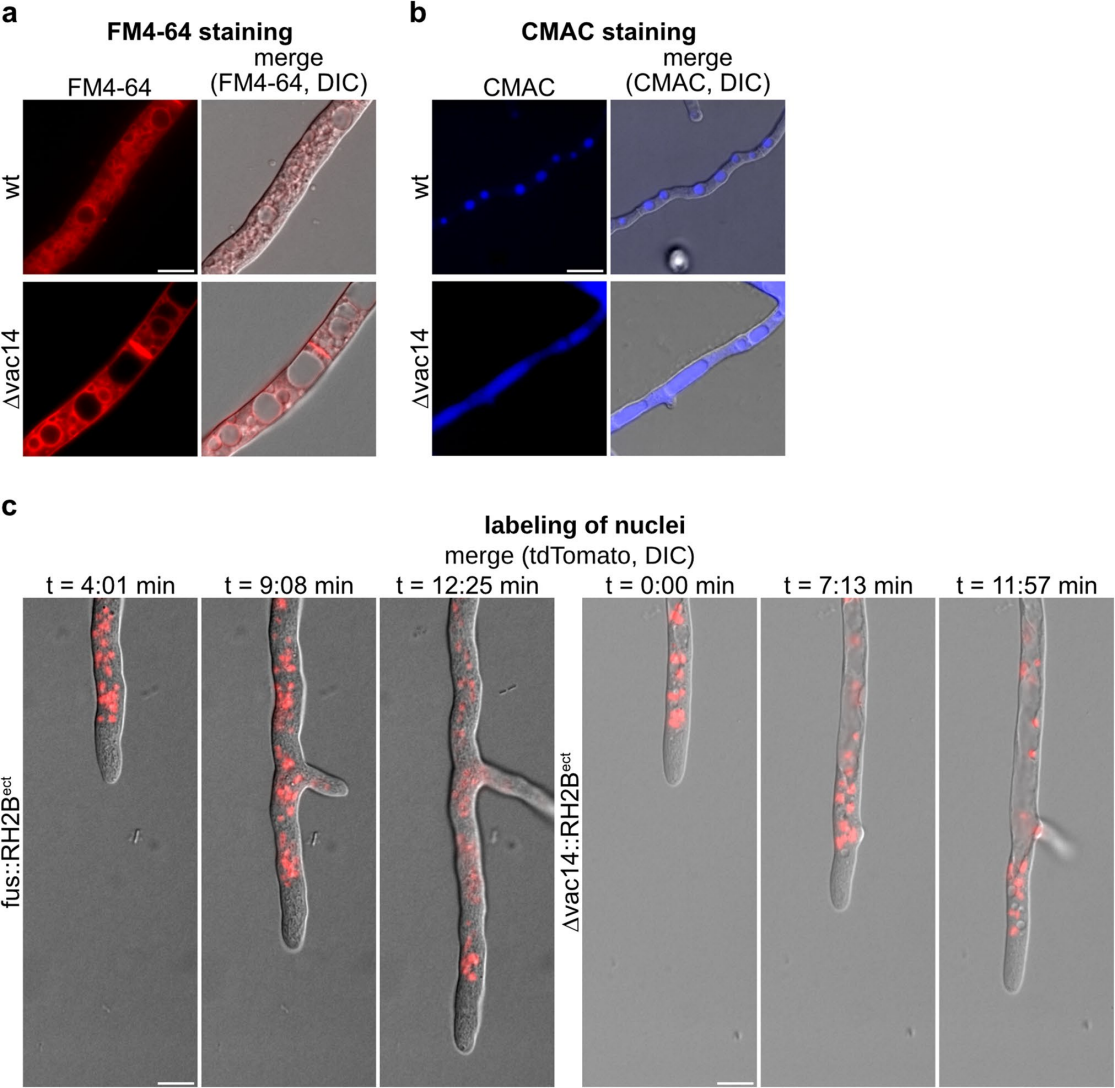
Vacuolar morphology of *S.* *macrospora* ∆vac14 and wt. **a** Vacuolar membranes of the hyphae were stained with FM4-64 (1 µg/mL in distilled water, and incubated for 15 min at 37 °C). Hyphae were recorded after growth on SWG + 1.5% agarose medium for 24 h at 27 °C under continuous light. **b** The lumen of the vacuoles was stained with CMAC (1:400 of 10 mM stock solution, and incubated for 30 min at 37 °C). Hyphae were recorded after growth over a piece of cellophane (0.5 cm × 0.5 cm) on solid SWG medium for 24 h at 27 °C under continuous light. **c** Selected images of Video S1 and Video S2 showing localization of enlarged vacuoles and distribution of nuclei in growing hyphae of the wt (S1) and ∆vac14 (S2) strain after 24 h on BMM + 1.5% agarose medium at 27 °C. Nuclei were labeled by histone 2B fused to tdTomato (RH2B). Scale bar = 10 µm, *DIC* differential interference contrast

Since sexual development and vegetative growth rate is impaired in the *S. macrospora* partial-deletion strain ∆vac14, we microscopically investigated living hyphae to analyze vacuolar morphology. Our results revealed an atypical morphology and appearance of vacuoles (Fig. 3). To visualize vacuolar membranes, we stained the *S.* *macrospora* wt and ∆vac14 strain with the red fluorescent and membrane-selective dye FM4-64 (Fischer-Parton et al. 2000; Penalva 2005) (Fig. 3a). For further staining of the acidic lumen of vacuoles, we used the fluorescent compound 7-amino-4-chloromethyl-coumarin (CMAC) (Cole et al. 1997, 1998) (Fig. 3b). These experiments revealed that the vacuoles of the ∆vac14 mutant were extremely enlarged when compared to the wt. Moreover, we examined the localization of nuclei in both strains at growing hyphal tips using the histone 2B labeled with tdTomato (RH2B) (Fig. 3c and Video S1 and Video S2). In the ∆vac14 strain, enlarged, cellular space-consuming vacuoles at hyphal tips appeared to displace nuclei to the periphery of the hyphae. This changed vacuolar morphology and distribution seemed to impair the growth rate of the ∆vac14 mutant in comparison to the wt (Fig. 3c).

### The ∆vac14 mutant is more stress sensitive to starvation stress than the wt

**Fig. 4:**
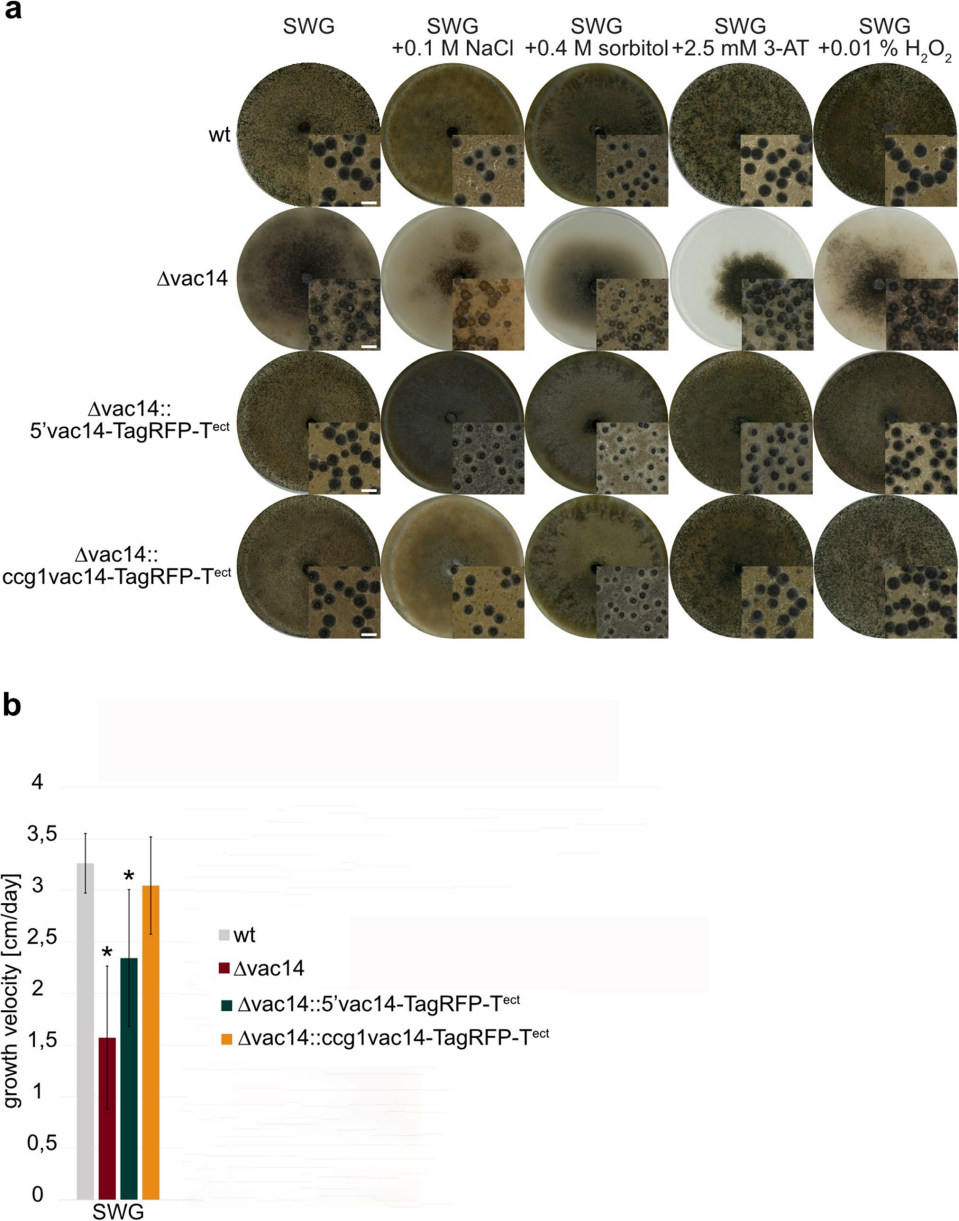
Sexual developmental on different stress media and vegetative growth rate of *S.* *macrospora* wt, ∆vac14 and the complementation strains ∆vac14::5’vac14-TagRFP-T^ect^ and ∆vac14::ccg1vac14-TagRFP-T^ect^. **a** Strains were grown in presence of various stress conditions, such as osmotic stress (0.1 M NaCl, 0.4 M sorbitol), under amino-acid starvation (2.5 mM 3-AT) or oxidative stress (0.01% H_2_O_2_) by adding the components to SWG medium. Pictures of the agar plates and enlargement of perithecia by microscopic images were taken after 10 days. Scale bar of microscopic images: 0.5 mm. **b** For determination of growth rate per day, strains were grown in 30-cm race tubes on SWG medium. Three biological replicates of each strain were analyzed in three independent experiments (*n* = 9). Asterisks (*) indicate a significant difference to the wt strain, according to Student’s t-test (*p* < 0.05)

Next, we analyzed the growth and developmental behavior of the ∆vac14 mutant when confronted with various stress conditions (Fig. 4). Sexual development of all strains was investigated after 10 days of growth on medium containing 0.1 M NaCl or 0.4 M sorbitol, mimicking osmotic stress, applying 2.5 mM 3-AT to generate amino-acid starvation or by adding 0.01% H_2_O_2_ for oxidative stress conditions (Fig. 4a). The wt, complementation, and vac14-overexpression strains were able to grow and form perithecia under all stress conditions and revealed normal sexual development. On the contrary, the ∆vac14 deletion strain displayed severe growth and developmental defects including decreased perithecia formation and slower growth rates (Fig. 4a).

Additionally, we tested the growth velocity in cm per day of all strains under normal and stress media (Fig. 4b and Fig. S7). The results revealed a significant growth impairment of the Δvac14 deletion mutant significant under normal growth conditions (SWG) (Fig. 4b). Significant growth impairment on media mimicking amino-acid starvation (2.5 mM 3-AT) were observed for all strains (Fig. S7). However, while growth of the wt was impaired by 29%, the growth of the ∆vac14 strains was reduced by 82% under starvation conditions (Fig. S7a and b). Further, osmotic stress conditions (0.1 M NaCl or 0.4 M sorbitol) also prevent normal sexual development in the ∆vac14 deletion mutant (Fig. 4a), but only 0.4 M sorbitol impaired the vegetative growth rate in comparison to normal growth conditions (Fig. S7b). Growth of ∆vac14 on oxidative stress (0.01% H_2_O_2_) lead to no further growth reduction as under normal conditions (SWG) (Fig. S7b). The complementation strains ∆vac14::5’vac14-TagRFP-T^ect^ and ∆vac14::ccg1vac14-TagRFP-T^ect^ showed similar development and growth under these conditions as the wt. However, overexpression of VAC14-TagRFP-T restored the ∆vac14 phenotype more efficiently (Fig. 4b, Fig. S7c and d). Limitation of nitrogen by omitting KNO_3_ from the medium and cell-wall stress by adding 0.003% SDS were also tested with no obvious effect concerning the development of the ∆vac14 strain (Fig. S8).

### SmVAC14 localizes to vacuolar membranes and to late endosomes

**Fig. 5:**
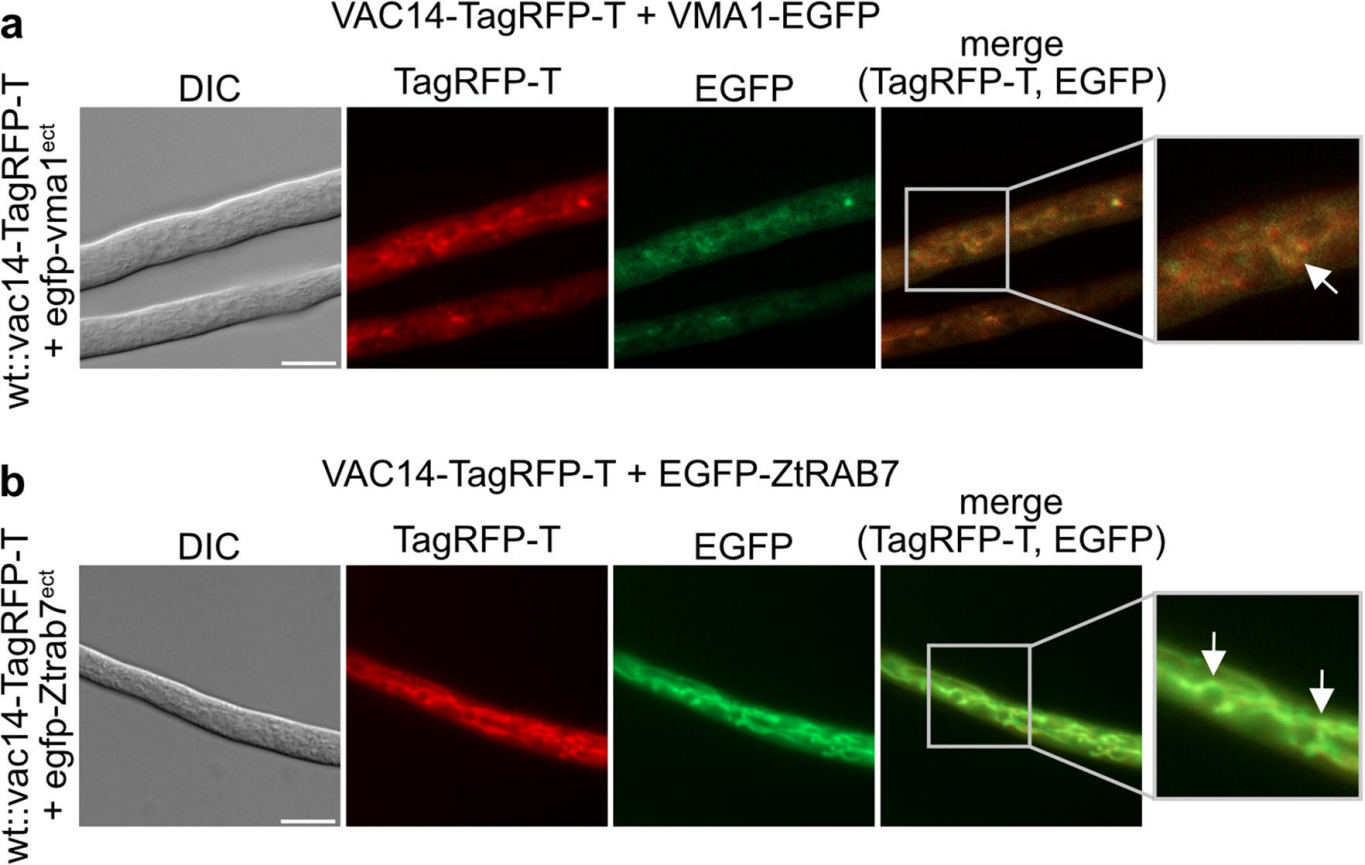
Co-localization of VAC14 and vacuolar and late endosomal marker proteins in apical hyphal compartments of the growth front using different fluorescence tags. *S.* *macrospora* wt were co-transformed and fluorescence microscopy was performed to visualize co-localization of the fusion proteins. **a** Co-transformed *S.* *macrospora* wt expressing VAC14-TagRFP-T and the tubular-vacuole marker VMA1 fused to EGFP. Co-localization of the fusion proteins is indicated by a white arrow. **b** *S.* *macrospora* wt expressing VAC14-TagRFP-T together with the fluorescence-tagged *Z.* *tritici* late endosomal marker EGFP-ZtRAB7. White arrows indicate co-localization of the fusion proteins. Scale bars = 10 µm, *DIC* differential interference contrast. Detailed twofold enlargements of the merge pictures are indicated by a frame and shown at the right margin

Fluorescence microscopy was performed to determine the subcellular localization of the *S. macrospora* VAC14 protein. SmVAC14 was C-terminally fused with TagRFP-T, to analyze the localization of VAC14 (Fig. 5). Due to the fact that the N-terminally tagged fusion protein TagRFP-T-VAC14 does not complement the ∆vac14 phenotype (Fig. S9), we performed the experiments with the C-terminally tagged protein version. Since the subcellular localization of VAC14 was not altered under the tested stress conditions (Fig. S10) and does not change whether it is expressed under the native (5') or the overexpression promotor of the *clock-controlled gene* 1 of *N. crassa* (*ccg1*) (Fig. S11), but the latter version resulted in increased fluorescence, we used this variant for fluorescence microscopy. For localization of free EGFP or TagRFP-T as control, *S.* *macrospora* wt and ∆vac14 strains were transformed with either plasmid p1783-1 (Pöggeler et al. 2003) or pDS23 (Teichert et al. 2012) or pTagRFP-T (Werner et al. 2021) (Fig. S12). To determine if VAC14 localizes with the highly dynamic vacuolar compartment, we generated a *S.* *macrospora* wt strain expressing VAC14-TagRFP-T and the vacuolar ATPase catalytic subunit A, VMA1, tagged with EGFP as reporter protein for vacuolar membranes and vesicles (Fig. 5a). Here, the merged picture indicated partial co-localization of VAC14-TagRFP-T with VMA1-EGFP at tubular shaped vacuoles and dot-like vesicular structures. Furthermore, we investigated if SmVAC14 also showed localization to the endocytic pathway by performing co-localization studies with the early- and late-endosomal reporter proteins of *Z.* *tritici* ZtRAB5 and ZtRAB7, respectively (Fig. S13a and Fig. 5b). Accordingly, VAC14-TagRFP-T appeared to co-localize, if at all, only partially with EGFP-ZtRAB5 at elongated filamentous structures (Fig. S13a). However, large rounded structures labeled by EGFP-ZtRAB5 do not co-localize to SmVAC14. It may, therefore, be that VAC14 only co-localize to a subpopulation of early endosomes. In contrast, VAC14-TagRFP-T showed distinct co-localization with EGFP-ZtRAB7 also at filamentous compartments and vesicles (Fig. 5b).

Localization of VAC14 at the ER was tested using the recently identified *S. macrospora* ER-marker protein POM33 (Groth et al. 2021). For this, *S.* *macrospora* wt strains expressing either VAC14-TagRFP-T or POM33-EGFP were crossed. Here, in the merged picture, no clear co-localization was observed (Fig. S13b). To analyze putative co-localization of VAC14 with the SmSTRIPAK-complex, *S.* *macrospora* wt was transformed with the plasmids pccg1vac14-TagRFP-T_hyg and p5’sci1-egfp (Reschka et al. 2018) to ectopically express the fusion proteins VAC14-TagRFP-T and SCI1-EGFP, respectively. Similar to POM33, no clear co-localization of VAC14 and SCI1 was observed (Fig. S13c).

To analyze the involvement of *S. macrospora vac14* in the endocytic pathway, we investigated the localization of the early endosomal marker EGFP-ZtRAB5 and the late endosomal marker EGFP-ZtRAB7 after 24 h and 72 h in the wt and the ∆vac14 deletion strain, respectively (Fig. S14). However, no clear effect on the localization of the endosomal marker proteins was observed.

### Autophagy is not affected by *vac14* deletion

**Fig. 6:**
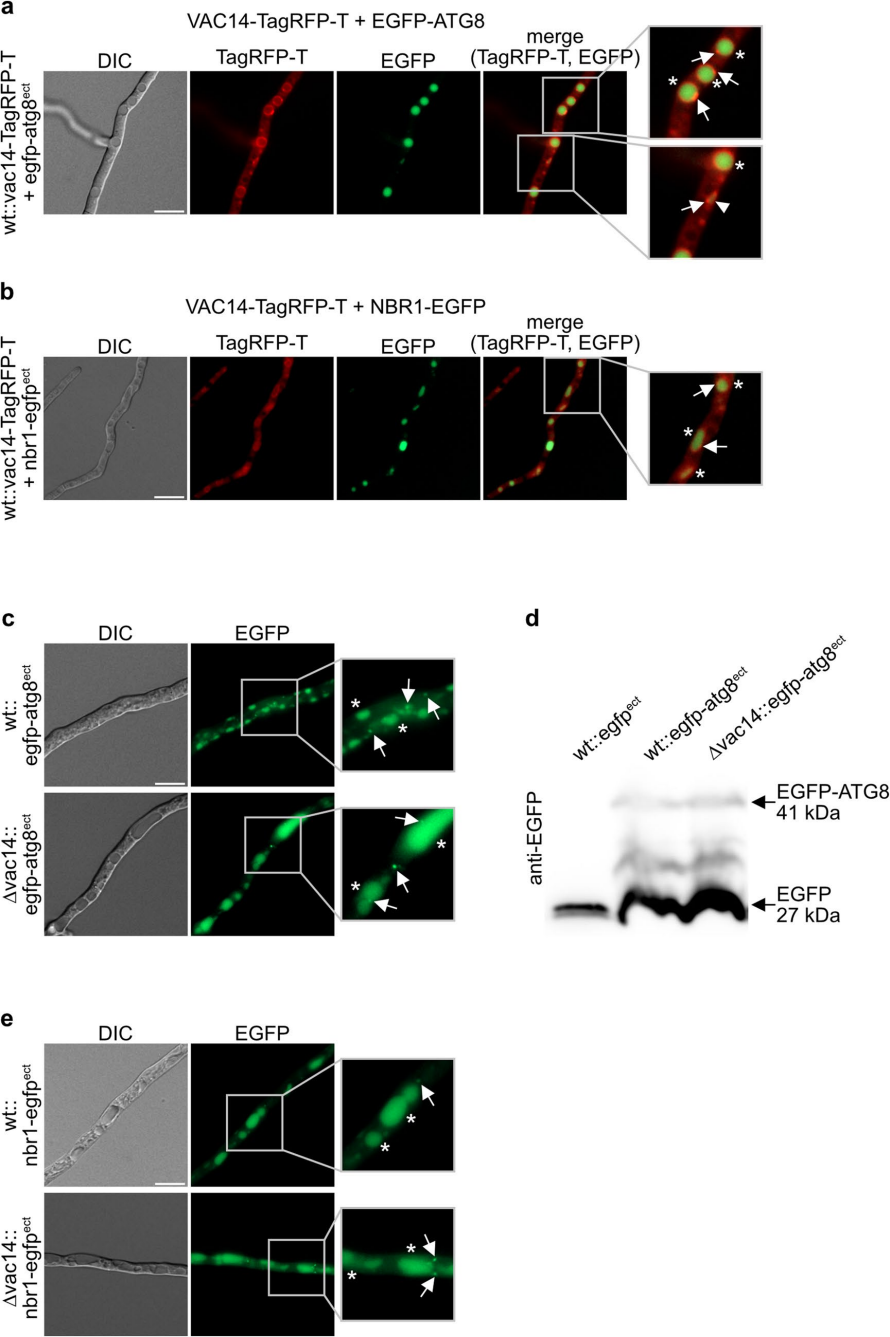
Co-localization of VAC14 with the autophagic marker proteins ATG8 and NBR1 in in sub-apical hyphal compartments *S.* *macrospora* wt and localization in ∆vac14. Strains were grown on solid SWG + 1.5% agarose medium for 72 h at 27 °C under continuous light conditions. *S.* *macrospora* wt expressing VAC14-TagRFP-T and EGFP-ATG 8 and VAC14-TagRFP-T and NBR1-EGFP, respectively, was used for fluorescence microscopy to visualize co-localization of fusion proteins (**a + b**) *S.* *macrospora* wt expressing VAC14-TagRFP-T together with the fluorescence-labeled autophagic marker EGFP-ATG8 (**a**) and the autophagy receptor NBR1-EGFP (**b**), respectively. After 72 h of growth the autophagic marker proteins displayed a localization inside of vacuoles marked by asterisks (*). White arrows indicate localization of VAC14-TagRFP-T around and at those vacuoles. A white arrowhead marks an autophagosome. Detailed twofold enlargements of the merge pictures are indicated by a frame and are shown at the right margin. *S.* *macrospora* wt and ∆vac14 strains expressing the fluorescence-labeled autophagy-marker proteins EGFP-ATG8 and NBR1-EGFP (**c–e**). The autophagic markers show localization inside vacuoles marked by asterisks (*). White arrows indicate localization in small dots, presumably autophagosomes. Detailed twofold enlargements of the merged pictures are indicated by a frame and shown at the right margin. **d** Western blot analysis for expression of EGFP-ATG8. The strain wt::egfp^ect^ served as control. Protein sizes are indicated. Degradation products of the fusion protein are visible. Scale bars = 10 µm, *DIC* differential interference contrast

Since our results revealed a strong stress-sensitive phenotype for ∆vac14 especially under amino-acid starvation conditions, we assumed that autophagy might be affected upon *Smvac14* deletion. In this context, it is noteworthy that deletion of *Smnbr1*, the autophagic receptor, also resulted in immature spore-formation (Werner et al. 2019), which is similar to the phenotype of ∆vac14. Moreover, human VAC14 was proposed to interact with endolysosomal and autophagic proteins (Schulze et al. 2014). First, we investigated the localization of SmVAC14 with autophagic marker proteins under non-starvation conditions. For this purpose, *S.* *macrospora* wt strains expressing VAC14-TagRFP-T together with either the EGFP-tagged autophagosomal-marker protein SmATG8 or the autophagic receptor SmNBR1, both fused to EGFP, were generated by crossing of the respected strains (Table 1) (Fig. 6a, b). To investigate the localization of these autophagy-marker proteins in ∆vac14, plasmids pegfp-atg8 (Voigt and Pöggeler 2013) and pnbr1-egfp (Werner et al. 2019), were transformed respectively. VAC14-TagRFP-T localized in the lumen as well as around vacuoles and at vacuolar compartments. The fusion proteins EGFP-ATG8 and NBR1-EGFP were degraded in the vacuole leading to stable green fluorescence in the vacuolar lumen (Fig. 6a, b). To further test if autophagy is affected upon *vac14* deletion, we performed fluorescence microscopy of both marker proteins in ∆vac14 and used Western blot analysis for degradation of EGFP-ATG8 (Fig. 6c–e). The results showed no alteration in the localizations of SmATG8 or SmNBR1 in the ∆vac14 deletion background compared to the wt (Fig. 6c, e). Moreover, SmATG8 was degraded similarly in ∆vac14 and wt, suggesting no effect of *vac14* deletion on autophagy.

## Discussion

The Fab1p/PIKfyve-multiprotein complex controls the generation of the minor phosphorylated phosphatidylinositol PtdIns(3,5)P_2_ and is comprised of the lipid and protein kinase Fab1p/PIKfyve, the lipid and protein phosphatase Fig4p /Sac3, and the scaffolding core Vac14p/ArPIKfyve. In yeast, additionally the Fab1p activator Vac7p and its inhibitor Atg18p are components of the Fab1p-complex (Bonangelino et al. 1997; Botelho et al. 2008; Duex et al. 2006b; Efe et al. 2007; Gary et al. 2002; Ikonomov et al. 2009a; Jin et al. 2008; Sbrissa et al. 2008; Schulze et al. 2014). In our study, we investigated the role of the Fab1p/PIKfyve scaffolding unit VAC14 of *S. macrospora*. We investigated the effect of *vac14* deletion and determined the subcellular localization of SmVAC14. Moreover, we analyzed the localization of endosomal and autophagy marker proteins in the ∆vac14 mutant strain.

Protein domain prediction revealed that SmVAC14 is a conserved protein that besides a Fab1/PIKfyve- and a Fig4/Sac3-binding domain and multiple TMDs as well as CCs is predicted to be composed of several ARM-repeats (Fig. 1). ARM- and HEAT-repeats are very similar evolutionary related motifs of tandemly repeated sequences of about 50 aa that provide surfaces for protein–protein interactions (Andrade et al. 2001; Cingolani et al. 1999; Malik et al. 1997). However, HEAT-repeats are degenerated and, therefore, difficult to be predicted by available online programs including the one we used here (Andrade et al. 2001). SmVAC14 together with Vac14 homologs in yeast in mammals can be classified as a member of the ARM-superfamily. Because the domain structure of SmVAC14 is similar to that of yeast, mammals and plants, a comparable function and localization can be assumed. To analyze the function of SmVAC14, a ∆vac14 deletion strain was generated and phenotypically investigated.

Accordingly, deletion of *Smvac14* caused deformed, less melanized perithecia and impaired ascospore formation (Fig. 2). Interestingly, in mammals the point mutant Vac14^L156R^, incapable of PIKfyve binding, induced the infantile gliosis (*ingls*) phenotype in mice characterized by less pigmentation and body size (Jin et al. 2008). Besides these phenotypic features, the *S. macrospora* ∆vac14 mutant also exhibited a high number of piled up perithecia around the inoculate agar piece compared to the remaining outer area of the petri dish (Fig. 2c). Fertile pile mutants were already described in *S. macrospora* showing defects in melanization of perithecia that are formed on top of each other; however, these mutants have not been molecularly analyzed (Engh et al. 2007; Kück et al. 2009; Teichert et al. 2014). Our studies showed that deletion of *Smvac14* resulted not only in impaired sexual development but also in enlarged vacuoles already present in growing hyphal tips (Figs. 2 and 3). A similar effect has been described for ∆fab1 and ∆vac14 deletion stains in yeast and mammals. Enlarged vacuoles and lysosomes were reported to be accompanied by loss of or lower levels of PtdIns(3,5)P_2_ (Gary et al. 1998; Ikonomov et al. 2001; Rusten et al. 2006; Yamamoto et al. 1995). In *S. cerevisiae*, fab1 mutants showed abnormal chromosome transmission, nuclear division and spindle morphology (Yamamoto et al. 1995). Similarly, we observed an ascospore defect in the Δvac14 mutant (Fig. S6). This defect might be caused by abnormal spindle morphology and nuclear division defects. PtdIns(3,5)P_2_ was recently proposed to activate the vacuolar (V)-ATPase H^ +^ -pump thereby maintaining sufficient acidification of the vacuoles and their morphology and size (Li et al. 2014). However, quantitative pH analysis revealed no defects in vacuolar acidification of *S. cerevisiae* ∆fab1p and ∆vac14p mutants (Ho et al. 2015). Moreover, PtdIns(3,5)P_2_ might not control the activity of the V-ATPase for steady-state conditions but rather in response to salt stress for osmoregulation (Li et al. 2014). Accordingly, in yeast regulation of the PtdIns(3,5)P_2_ level at vacuolar membranes is controlled by hyperosmotic stress (Bonangelino et al. 2002; Jin et al. 2017). Therefore, a disturbed osmotic regulation was assumed to cause enlargement of vacuoles and endolysosomes (Banerjee and Kane 2020; Wilson et al. 2018).

Similarly, the *S. macrospora* ∆vac14 strain revealed a strong stress-phenotype by being hypersensitive to sorbitol stress and amino-acid starvation stress conditions (Fig. 4 and Fig. S7). This observation is consistent with those in yeast, where ∆fab1, ∆fig4 and ∆vac14 mutants also reacted to hyperosmotic shock by increased PtdIns(3,5)P_2_ levels that returned to its native quantity quickly after stressing the cells (Bonangelino et al. 2002; Dove et al. 1997; Duex et al. 2006b).

For localization studies of SmVAC14, we performed fluorescence microscopy. Recently, pulldown experiments had identified SmVAC14 as potential interaction partner of SCI1 (Reschka et al. 2018). Thus, connection between both proteins had been proposed, which was although, not clearly confirmed by the co-localization of both proteins in our studies (Fig. S13c). However, other recent findings indicate a possible connection of VAC14 with the STRIPAK-complex. Global phospho-proteomic studies in *S. macrospora* revealed that SmVAC14 is differentially phosphorylated at T455 and S429 in SmSTRIPAK mutants, suggesting a link between the scaffolding protein SmVAC14 and the SmSTRIPAK-complex (Märker et al. 2020; Stein et al. 2020). Moreover, a recently performed proximity-dependent biotin identification (BioID) analysis with mammalian Vac14 and Fig4 revealed STRIPAK-components in proximity as potential interactors (Qiu et al. 2021).

Furthermore, fluorescence microscopy revealed that SmVAC14 localized at vacuolar membranes and with late endosomes (Fig. 5). These results are concurrent with observations in yeast and mammals, where Vac14p/ArPIKfyve localizes to the membranes of vacuoles and to endolysosomes, respectively (Bonangelino et al. 2002; Dove et al. 2002; Jin et al. 2008).

In mammals, enlarged vacuoles of Vac14^wt^ or PIKfyve-binding deficient Vac14^L156R^ overexpression cells were shown to be positive for the late endosomal markers Rab7, CD63, and Lamp2 (Schulze et al. 2014, 2017). In contrast to this, highly enlarged vacuolar/endolysosomal compartments could not be detected when *Smvac14* was overexpressed in *S. macrospora* (Fig. S11).

Because proteins that are linked to lysosomal and autophagic membrane dynamics (Rab9, Rab7 activator TBC1D15, and Rab5-interacting protein Sun2) were identified as potential interaction partners of mammalian Vac14 (Schulze et al. 2014, 2017), we investigated if autophagy might be affected in the *S. macrospora* ∆vac14 deletion strain (Fig. 6a, b). SmVAC14 localized at membranes of vacuoles and autophagosomes, but autophagy was apparently not affected in ∆vac14. These observations are consistent with those of mammalian Vac14 overexpression mutants, where the autophagic flux was not affected by enlarged lysosomes, although LC3 (ATG8) accumulated in Western blot analysis of these mutants (Schulze et al. 2014, 2017). In addition, in *ingls* mice, as well as in *Drosophila melanogaster* and *Caenorhabditis elegans vac14* deletion mutants accumulation of autophagosomes was detected (de Lartigue et al. 2009; Ferguson et al. 2009; Ho et al. 2012; Nicot et al. 2006; Rusten et al. 2007).

To obtain an overview of the diverse functions of the Fab1/PIKfyve-complex and its potential connection with other regulatory complexes and signaling pathways in *S. macrospora* and to unravel mechanistic links between VAC14, PtdIns(3,5)P_2_ homeostasis and developmental processes in filamentous fungi further studies are required.

## Supplementary Information

Below is the link to the electronic supplementary material.

Supplementary file1 (PDF 4631 KB)

Supplementary file2 (AVI 4497 KB)

Supplementary file3 (AVI 5067 KB)

## References

[CR1] Andrade MA, Petosa C, O'Donoghue SI, Muller CW, Bork P (2001). Comparison of ARM and HEAT protein repeats. J Mol Biol.

[CR2] Banerjee S, Kane PM (2020). Regulation of V-ATPase activity and organelle pH by phosphatidylinositol phosphate lipids. Front Cell Dev Biol.

[CR3] Beier A, Teichert I, Krisp C, Wolters DA, Kück U (2016). Catalytic subunit 1 of protein phosphatase 2A is a subunit of the STRIPAK complex and governs fungal sexual development. Mbio.

[CR4] Bernhards Y, Pöggeler S (2011). The phocein homologue SmMOB3 is essential for vegetative cell fusion and sexual development in the filamentous ascomycete *Sordaria macrospora*. Curr Genet.

[CR5] Blank-Landeshammer B, Teichert I, Märker R, Nowrousian M, Kück U, Sickmann A (2019). Combination of proteogenomics with peptide de novo sequencing identifies new genes and hidden posttranscriptional modifications. mBio.

[CR6] Bloemendal S, Bernhards Y, Bartho K, Dettmann A, Voigt O, Teichert I, Seiler S, Wolters DA, Pöggeler S, Kück U (2012). A homologue of the human STRIPAK complex controls sexual development in fungi. Mol Microbiol.

[CR7] Blum M, Chang HY, Chuguransky S, Grego T, Kandasaamy S, Mitchell A, Nuka G, Paysan-Lafosse T, Qureshi M, Raj S, Richardson L, Salazar GA, Williams L, Bork P, Bridge A, Gough J, Haft DH, Letunic I, Marchler-Bauer A, Mi H, Natale DA, Necci M, Orengo CA, Pandurangan AP, Rivoire C, Sigrist CJA, Sillitoe I, Thanki N, Thomas PD, Tosatto SCE, Wu CH, Bateman A, Finn RD (2021). The InterPro protein families and domains database: 20 years on. Nucleic Acids Res.

[CR8] Bonangelino CJ, Catlett NL, Weisman LS (1997). Vac7p, a novel vacuolar protein, is required for normal vacuole inheritance and morphology. Mol Cell Biol.

[CR9] Bonangelino CJ, Nau JJ, Duex JE, Brinkman M, Wurmser AE, Gary JD, Emr SD, Weisman LS (2002). Osmotic stress-induced increase of phosphatidylinositol 3,5-bisphosphate requires Vac14p, an activator of the lipid kinase Fab1p. J Cell Biol.

[CR10] Botelho RJ, Efe JA, Teis D, Emr SD (2008). Assembly of a Fab1 phosphoinositide kinase signaling complex requires the Fig4 phosphoinositide phosphatase. Mol Biol Cell.

[CR11] Chow CY, Zhang Y, Dowling JJ, Jin N, Adamska M, Shiga K, Szigeti K, Shy ME, Li J, Zhang X, Lupski JR, Weisman LS, Meisler MH (2007). Mutation of FIG4 causes neurodegeneration in the pale tremor mouse and patients with CMT4J. Nature.

[CR12] Chow CY, Landers JE, Bergren SK, Sapp PC, Grant AE, Jones JM, Everett L, Lenk GM, McKenna-Yasek DM, Weisman LS, Figlewicz D, Brown RH, Meisler MH (2009). Deleterious variants of FIG4, a phosphoinositide phosphatase, in patients with ALS. Am J Hum Genet.

[CR13] Cingolani G, Petosa C, Weis K, Muller CW (1999). Structure of importin-beta bound to the IBB domain of importin-alpha. Nature.

[CR14] Cole L, Hyde G, Ashford A (1997). Uptake and compartmentalisation of fluorescent probes by *Pisolithus tinctorius* hyphae: evidence for an anion transport mechanism at the tonoplast but not for fluid-phase endocytosis. Protoplasma.

[CR15] Cole L, Orlovich DA, Ashford AE (1998). Structure, function, and motility of vacuoles in filamentous fungi. Fungal Genet Biol.

[CR16] Colot HV, Park G, Turner GE, Ringelberg C, Crew CM, Litvinkova L, Weiss RL, Borkovich KA, Dunlap JC (2006). A high-throughput gene knockout procedure for Neurospora reveals functions for multiple transcription factors. Proc Natl Acad Sci U S A.

[CR17] Dahlmann TA, Terfehr D, Becker K, Teichert I (2021). Golden Gate vectors for efficient gene fusion and gene deletion in diverse filamentous fungi. Curr Genet.

[CR18] de Lartigue J, Polson H, Feldman M, Shokat K, Tooze SA, Urbe S, Clague MJ (2009). PIKfyve regulation of endosome-linked pathways. Traffic.

[CR19] Dove SK, Cooke FT, Douglas MR, Sayers LG, Parker PJ, Michell RH (1997). Osmotic stress activates phosphatidylinositol-3,5-bisphosphate synthesis. Nature.

[CR20] Dove SK, McEwen RK, Mayes A, Hughes DC, Beggs JD, Michell RH (2002). Vac14 controls PtdIns(3,5)P(2) synthesis and Fab1-dependent protein trafficking to the multivesicular body. Curr Biol.

[CR21] Dove SK, Dong K, Kobayashi T, Williams FK, Michell RH (2009). Phosphatidylinositol 3,5-bisphosphate and Fab1p/PIKfyve underPPIn endo-lysosome function. Biochem J.

[CR22] Duex JE, Nau JJ, Kauffman EJ, Weisman LS (2006). Phosphoinositide 5-phosphatase Fig 4p is required for both acute rise and subsequent fall in stress-induced phosphatidylinositol 3,5-bisphosphate levels. Eukaryot Cell.

[CR23] Duex JE, Tang F, Weisman LS (2006). The Vac14p-Fig4p complex acts independently of Vac7p and couples PI3,5P2 synthesis and turnover. J Cell Biol.

[CR24] Efe JA, Botelho RJ, Emr SD (2005). The Fab1 phosphatidylinositol kinase pathway in the regulation of vacuole morphology. Curr Opin Cell Biol.

[CR25] Efe JA, Botelho RJ, Emr SD (2007). Atg18 regulates organelle morphology and Fab1 kinase activity independent of its membrane recruitment by phosphatidylinositol 3,5-bisphosphate. Mol Biol Cell.

[CR26] Elleuche S, Pöggeler S (2009). Evolution of carbonic anhydrases in fungi. Curr Genet.

[CR27] Engh I, Nowrousian M, Kück U (2007). Regulation of melanin biosynthesis via the dihydroxynaphthalene pathway is dependent on sexual development in the ascomycete *Sordaria macrospora*. FEMS Microbiol Lett.

[CR28] Esser K, Straub J (1958). Genetic studies on Sordaria macrospora Auersw, compensation and induction in gene-dependent developmental defects. Z Vererbungsl.

[CR29] Esser K 1982 Cryptogams: cyanobacteria, algae, fungi, lichens CUP archive

[CR30] Ferguson CJ, Lenk GM, Meisler MH (2009). Defective autophagy in neurons and astrocytes from mice deficient in PI(3,5)P2. Hum Mol Genet.

[CR31] Fischer-Parton S, Parton RM, Hickey PC, Dijksterhuis J, Atkinson HA, Read ND (2000). Confocal microscopy of FM4-64 as a tool for analysing endocytosis and vesicle trafficking in living fungal hyphae. J Microsc.

[CR32] Frey S, Reschka EJ, Pöggeler S (2015). Germinal center kinases SmKIN3 and SmKIN24 are associated with the *Sordaria macrospora* striatin-interacting phosphatase and kinase (STRIPAK) Complex. PLoS ONE.

[CR33] Gary JD, Wurmser AE, Bonangelino CJ, Weisman LS, Emr SD (1998). Fab1p is essential for PtdIns(3)P 5-kinase activity and the maintenance of vacuolar size and membrane homeostasis. J Cell Biol.

[CR34] Gary JD, Sato TK, Stefan CJ, Bonangelino CJ, Weisman LS, Emr SD (2002). Regulation of Fab1 phosphatidylinositol 3-phosphate 5-kinase pathway by Vac7 protein and Fig4, a polyphosphoinositide phosphatase family member. Mol Biol Cell.

[CR35] Groth A, Schmitt K, Valerius O, Herzog B, Pöggeler S (2021). Analysis of the putative nucleoporin POM33 in the filamentous fungus *Sordaria macrospora*. J Fungi.

[CR36] Ho CY, Alghamdi TA, Botelho RJ (2012). Phosphatidylinositol-3,5-bisphosphate: no longer the poor PIP2. Traffic.

[CR37] Ho CY, Choy CH, Wattson CA, Johnson DE, Botelho RJ (2015). The Fab1/PIKfyve phosphoinositide phosphate kinase is not necessary to maintain the pH of lysosomes and of the yeast vacuole. J Biol Chem.

[CR38] Hwang J, Pallas DC (2014). STRIPAK complexes: structure, biological function, and involvement in human diseases. Int J Biochem Cell Biol.

[CR39] Ikonomov OC, Sbrissa D, Shisheva A (2001). Mammalian cell morphology and endocytic membrane homeostasis require enzymatically active phosphoinositide 5-kinase PIKfyve. J Biol Chem.

[CR40] Ikonomov OC, Sbrissa D, Fenner H, Shisheva A (2009). PIKfyve-ArPIKfyve-Sac3 core complex: contact sites and their consequence for Sac3 phosphatase activity and endocytic membrane homeostasis. J Biol Chem.

[CR41] Ikonomov OC, Sbrissa D, Ijuin T, Takenawa T, Shisheva A (2009). Sac3 is an insulin-regulated phosphatidylinositol 3,5-bisphosphate phosphatase: gain in insulin responsiveness through Sac3 down-regulation in adipocytes. J Biol Chem.

[CR42] James P, Halladay J, Craig EA (1996). Genomic libraries and a host strain designed for highly efficient two-hybrid selection in yeast. Genetics.

[CR43] Jin N, Chow CY, Liu L, Zolov SN, Bronson R, Davisson M, Petersen JL, Zhang Y, Park S, Duex JE, Goldowitz D, Meisler MH, Weisman LS (2008). VAC14 nucleates a protein complex essential for the acute interconversion of PI3P and PI(3,5)P(2) in yeast and mouse. EMBO J.

[CR44] Jin N, Jin Y, Weisman LS (2017). Early protection to stress mediated by CDK-dependent PI3,5P2 signaling from the vacuole/lysosome. J Cell Biol.

[CR45] Katoh K, Rozewicki J, Yamada KD (2019). MAFFT online service: multiple sequence alignment, interactive sequence choice and visualization. Brief Bioinform.

[CR46] Kilaru S, Schuster M, Latz M, Guo M, Steinberg G (2015). Fluorescent markers of the endocytic pathway in *Zymoseptoria tritici*. Fungal Genet Biol.

[CR47] Klix V, Nowrousian M, Ringelberg C, Loros JJ, Dunlap JC, Pöggeler S (2010). Functional characterization of MAT1-1-specific mating-type genes in the homothallic ascomycete Sordaria macrospora provides new insights into essential and nonessential sexual regulators. Eukaryot Cell.

[CR48] Kück U, Hoff B (2006). Application of the nourseothricin acetyltransferase gene (nat1) as dominant marker for the transformation of filamentous fungi. Fungal Genet Newsl.

[CR49] Kück U, Pöggeler S, Nowrousian M, Nolting N, Engh I (2009). *Sordaria macrospora*, a model system for fungal development physiology and genetics.

[CR50] Kück U, Beier AM, Teichert I (2016). The composition and function of the striatin-interacting phosphatases and kinases (STRIPAK) complex in fungi. Fungal Genet Biol.

[CR51] Kück U, Radchenko D, Teichert I (2019). STRIPAK, a highly conserved signaling complex, controls multiple eukaryotic cellular and developmental processes and is linked with human diseases. Biol Chem.

[CR52] Lemaire JF, McPherson PS (2006). Binding of Vac14 to neuronal nitric oxide synthase: characterisation of a new internal PDZ-recognition motif. FEBS Lett.

[CR53] Li SC, Diakov TT, Xu T, Tarsio M, Zhu W, Couoh-Cardel S, Weisman LS, Kane PM (2014). The signaling lipid PI(3,5)P(2) stabilizes V(1)-V(o) sector interactions and activates the V-ATPase. Mol Biol Cell.

[CR54] Lupas A, Van Dyke M, Stock J (1991). Predicting coiled coils from protein sequences. Science.

[CR55] Malik HS, Eickbush TH, Goldfarb DS (1997). Evolutionary specialization of the nuclear targeting apparatus. Proc Natl Acad Sci U S A.

[CR56] Märker R, Blank-Landeshammer B, Beier-Rosberger A, Sickmann A, Kück U (2020). Phosphoproteomic analysis of STRIPAK mutants identifies a conserved serine phosphorylation site in PAK kinase CLA4 to be important in fungal sexual development and polarized growth. Mol Microbiol.

[CR57] Nicholas KB, Nicholas H (1997) GeneDoc: a tool for editing and annoting multiple sequence alignments.

[CR58] Nicot AS, Fares H, Payrastre B, Chisholm AD, Labouesse M, Laporte J (2006). The phosphoinositide kinase PIKfyve/Fab1p regulates terminal lysosome maturation in *Caenorhabditis elegans*. Mol Biol Cell.

[CR59] Nowrousian M, Ringelberg C, Dunlap JC, Loros JJ, Kück U (2005). Cross-species microarray hybridization to identify developmentally regulated genes in the filamentous fungus *Sordaria macrospora*. Mol Genet Genom.

[CR60] Nowrousian M, Teichert I, Masloff S, Kück U (2012). Whole-genome sequencing of *Sordaria macrospora* mutants identifies developmental genes. G3.

[CR61] Penalva MA (2005). Tracing the endocytic pathway of *Aspergillus nidulans* with FM4-64. Fungal Genet Biol.

[CR62] Pöggeler S, Kück U (2004). A WD40 repeat protein regulates fungal cell differentiation and can be replaced functionally by the mammalian homologue striatin. Eukaryot Cell.

[CR63] Pöggeler S, Kück U (2006). Highly efficient generation of signal transduction knockout mutants using a fungal strain deficient in the mammalian ku70 ortholog. Gene.

[CR64] Pöggeler S, Masloff S, Hoff B, Mayrhofer S, Kück U (2003). Versatile EGFP reporter plasmids for cellular localization of recombinant gene products in filamentous fungi. Curr Genet.

[CR65] Pöggeler S, Nowrousian M, Kück U (2006). Fruiting-body development in ascomycetes growth, differentiation and sexuality.

[CR66] Qiu S, Lavallee-Adam M, Cote M (2021). Proximity interactome map of the Vac14-Fig4 complex using BioID. J Proteome Res.

[CR67] Reschka EJ, Nordzieke S, Valerius O, Braus GH, Pöggeler S (2018). A novel STRIPAK complex component mediates hyphal fusion and fruiting-body development in filamentous fungi. Mol Microbiol.

[CR68] Robinson O, Dylus D, Dessimoz C (2016). Phylo.io: interactive viewing and comparison of large phylogenetic trees on the web. Mol Biol Evol.

[CR69] Rudge SA, Anderson DM, Emr SD (2004). Vacuole size control: regulation of PtdIns(3,5)P2 levels by the vacuole-associated Vac14-Fig4 complex, a PtdIns(3,5)P2-specific phosphatase. Mol Biol Cell.

[CR70] Rusten TE, Rodahl LM, Pattni K, Englund C, Samakovlis C, Dove S, Brech A, Stenmark H (2006). Fab1 phosphatidylinositol 3-phosphate 5-kinase controls trafficking but not silencing of endocytosed receptors. Mol Biol Cell.

[CR71] Rusten TE, Vaccari T, Lindmo K, Rodahl LM, Nezis IP, Sem-Jacobsen C, Wendler F, Vincent JP, Brech A, Bilder D, Stenmark H (2007). ESCRTs and Fab1 regulate distinct steps of autophagy. Curr Biol.

[CR72] Rutherford AC, Traer C, Wassmer T, Pattni K, Bujny MV, Carlton JG, Stenmark H, Cullen PJ (2006). The mammalian phosphatidylinositol 3-phosphate 5-kinase (PIKfyve) regulates endosome-to-TGN retrograde transport. J Cell Sci.

[CR73] Sambrook J, Fritsch E, Maniatis T (2001) Molecular cloning: a laboratory manual. Cold Spring Harbor, New York.

[CR74] Sbrissa D, Ikonomov OC, Strakova J, Dondapati R, Mlak K, Deeb R, Silver R, Shisheva A (2004). A mammalian ortholog of Saccharomyces cerevisiae Vac14 that associates with and up-regulates PIKfyve phosphoinositide 5-kinase activity. Mol Cell Biol.

[CR75] Sbrissa D, Ikonomov OC, Fu Z, Ijuin T, Gruenberg J, Takenawa T, Shisheva A (2007). Core protein machinery for mammalian phosphatidylinositol 3,5-bisphosphate synthesis and turnover that regulates the progression of endosomal transport. Novel Sac phosphatase joins the ArPIKfyve-PIKfyve complex. J Biol Chem.

[CR76] Sbrissa D, Ikonomov OC, Fenner H, Shisheva A (2008). ArPIKfyve homomeric and heteromeric interactions scaffold PIKfyve and Sac3 in a complex to promote PIKfyve activity and functionality. J Mol Biol.

[CR77] Schulze U, Vollenbroker B, Braun DA, Van Le T, Granado D, Kremerskothen J, Franzel B, Klosowski R, Barth J, Fufezan C, Wolters DA, Pavenstadt H, Weide T (2014). The Vac14-interaction network is linked to regulators of the endolysosomal and autophagic pathway. Mol Cell Proteomics.

[CR78] Schulze U, Vollenbröker B, Kuhnl A, Granado D, Bayraktar S, Rescher U, Pavenstadt H, Weide T (2017). Cellular vacuolization caused by overexpression of the PIKfyve-binding deficient Vac 14(L156R) is rescued by starvation and inhibition of vacuolar-ATPase. Biochim Biophys Acta Mol Cell Res.

[CR79] Shi Z, Jiao S, Zhou Z (2016). STRIPAK complexes in cell signaling and cancer. Oncogene.

[CR80] Shisheva A (2008). PIKfyve: Partners, significance, debates and paradoxes. Cell Biol Int.

[CR81] Stein V, Blank-Landeshammer B, Muntjes K, Märker R, Teichert I, Feldbrugge M, Sickmann A, Kück U (2020). The STRIPAK signaling complex regulates dephosphorylation of GUL1, an RNA-binding protein that shuttles on endosomes. PLoS Genet.

[CR82] Teichert I, Wolff G, Kück U, Nowrousian M (2012). Combining laser microdissection and RNA-seq to chart the transcriptional landscape of fungal development. BMC Genomics.

[CR83] Teichert I, Nowrousian M, Pöggeler S, Kück U (2014). The filamentous fungus *Sordaria macrospora* as a genetic model to study fruiting body development. Adv Genet.

[CR84] Teichert I, Pöggeler S, Nowrousian M (2020). *Sordaria macrospora*: 25 years as a model organism for studying the molecular mechanisms of fruiting body development. Appl Microbiol Biotechnol.

[CR85] Towbin H, Staehelin T, Gordon J (1979). Electrophoretic transfer of proteins from polyacrylamide gels to nitrocellulose sheets: procedure and some applications. Proc Natl Acad Sci U S A.

[CR86] Tusnády GE, Simon I (2001). The HMMTOP transmembrane topology prediction server. Bioinformatics.

[CR87] Vicinanza M, D'Angelo G, Di Campli A, De Matteis MA (2008). Function and dysfunction of the PI system in membrane trafficking. EMBO J.

[CR88] Voigt O, Pöggeler S (2013). Autophagy genes Smatg8 and Smatg4 are required for fruiting-body development, vegetative growth and ascospore germination in the filamentous ascomycete *Sordaria macrospora*. Autophagy.

[CR89] Walz M, Kück U (1995). Transformation of *Sordaria macrospora* to hygromycin B resistance: characterization of transformants by electrophoretic karyotyping and tetrad analysis. Curr Genet.

[CR92] Werner A (2012) Functional analysis of the putative autophagy receptor SmNBR1 and the autophagic protein SmATG12 of the filamentous ascomycete *Sordaria macrospora* Master thesis. Georg-August Univerität Göttingen.

[CR90] Werner A, Herzog B, Voigt O, Valerius O, Braus GH, Pöggeler S (2019). NBR1 is involved in selective pexophagy in filamentous ascomycetes and can be functionally replaced by a tagged version of its human homolog. Autophagy.

[CR91] Werner A, Otte K, Stahlhut G, Hanke LM, Pöggeler S (2021). The glyoxysomal protease LON2 is involved in fruiting-body development, ascosporogenesis and stress resistance in *Sordaria macrospora*. J Fungi.

[CR93] Wilson ZN, Scott AL, Dowell RD, Odorizzi G (2018). PI(3,5)P2 controls vacuole potassium transport to support cellular osmoregulation. Mol Biol Cell.

[CR94] Yamamoto A, DeWald BD, Boronenkov IV, Anderson RA, Emr SD, Koshland D (1995). Novel PI(4)P 5-kinase homologue, Fab1p, essential for normal vacuole function and morphology in yeast. Mol Biol Cell.

[CR95] Zhang Y, Zolov SN, Chow CY, Slutsky SG, Richardson SC, Piper RC, Yang B, Nau JJ, Westrick RJ, Morrison SJ, Meisler MH, Weisman LS (2007). Loss of Vac14, a regulator of the signaling lipid phosphatidylinositol 3,5-bisphosphate, results in neurodegeneration in mice. Proc Natl Acad Sci U S A.

[CR96] Zhang X, Chow CY, Sahenk Z, Shy ME, Meisler MH, Li J (2008). Mutation of FIG4 causes a rapidly progressive, asymmetric neuronal degeneration. Brain.

