# Supplementary material for: The vacuolar morphology protein VAC14 plays an important role in sexual development in the filamentous ascomycete Sordaria macrospora
Source: Curr Genet. 2022 Jul 1;68(3-4):407–27. doi: 10.1007/s00294-022-01244-0 (PMC9279277; doi:10.1007/s00294-022-01244-0)
Supplement: Supplementary file 1 — Supplementary file1 (PDF 4631 KB) [file 294_2022_1244_MOESM1_ESM.pdf]

The Vacuolar Morphology Protein VAC14 Plays an Important Role in Sexual Development in the Filamentous Ascomycete *Sordaria macrospora*

Anika Groth [1], Svenja Ahlmann [1], Antonia Werner [1] and Stefanie Pöggeler [1,*]

[1] Department of Genetics of Eukaryotic Microorganisms, Institute of Microbiology and Genetics, Georg-August-University of Göttingen, Grisebachstr. 8, 37077 Göttingen, Germany; anika.gibron@uni-goettingen.de (A.G.); svenja.ahlmann@stud.uni-goettingen.de (S.A.); ajakobs1@gwdg.de (A.W.)

[*] Correspondence: spoegge@gwdg.de; Tel.: +49-551-39-24051

Tab. S1 List of plasmids used and generated in this study

Plasmid	Feature	Reference
pRS-nat	*amp*^{R}, *ura3*, *nat*^{R}	(Klix et al. 2010)
pRS-hyg	*amp*^{R}, *ura3*, *hyg*^{R}	(Bloemendal et al. 2012)
pJet_nat	*amp*^{R}, *nat*^{R}	Nordzieke, unpublished
p1783-1	*amp*^{R}, *ura3*, *hyg*^{R}<br>*Pgpd::egfp::TtrpC*	(Pöggeler et al. 2003)
pDS23	*amp*^{R}, *ura3*, *nat*^{R}<br>*Pgpd::egfp::TtrpC*	(Teichert et al. 2012)
pTagRFP-T	*amp*^{R}, *nat*^{R}<br>*Pccg1::TagRFP-T::TtrpC*	(Werner et al. 2021)
pGG-hph	*kan*^{R}, *bla*<br>*BsaI(6)::PtrpC::hph::BsaI(5)* in pDrive	(Dahlmann et al. 2021)
pDest-Amp	Destination vector for Golden Gate cloning; *bla* (Bsamut), *lacZ* gene with two internal *BsaI* sites: *BsaI(4)* and *BsaI(7)*	(Dahlmann et al. 2021)
pRH2B_nat	*amp*^{R}, *ura3*, *nat*^{R}<br>*Pgpd::hh2b::tdTomato::TtrpC*	(Reschka et al. 2018)
pHeGFPRab5_hyg	*kan*^{R}, *ura3*, *hyg*^{R}<br>*PZttub2::egfp::Ztrab5::TZttub2*	(Kilaru et al. 2015)
pHeGFPRab7_hyg	*kan*^{R}, *ura3*, *hyg*^{R}<br>*PZttub2::egfp::Ztrab7::TZttub2*	(Kilaru et al. 2015)
pnbr1-egfp	*amp*^{R}, *ura3*, *nat*^{R}<br>*Pnbr1::nbr1::egfp::TtrpC*	(Werner et al. 2019)
pegfp-atg8	*nat*^{R}<br>*Patg8::egfp::atg8::Tatg8*	(Voigt and Pöggeler 2013)
p5'sci1-egfp	*amp*^{R}, *ura3*, *nat*^{R}<br>*Psci1::sci1::egfp::TtrpC*	(Reschka et al. 2018)
pegfp-Ztrab5_nat	*amp*^{R}, *ura3*, *nat*^{R}<br>*PZttub2::egfp::Ztrab5::TZttub2*	This study
pegfp-Ztrab7_nat	*amp*^{R}, *ura3*, *nat*^{R}<br>*PZttub2::egfp::Ztrab7::TZttub2*	This study
pegfp-Ztrab5_hyg	*amp*^{R}, *ura3*, hyg^R<br>*PZttub2::egfp::Ztrab5::TZttub2*	This study
pegfp-Ztrab7_hyg	*amp*^{R}, *ura3*, hyg^R<br>*PZttub2::egfp::Ztrab7::TZttub2*	This study
pegfp-vma1	*amp*^{R}, *ura3*, *hyg*^{R}<br>*Pvma1::egfp::vma1::Tvma1*	This study
pvac14-KO_V3w	*amp*^{R}, first 1030bp (=5'-flank) and last 1030bp (=3'-flank) of *vac14* ORF interrupted by *hyg*^{R} in pDest-Amp	This study
p5'vac14-TagRFP-T	*amp*^{R}, *ura3*, *nat*^{R}<br>*Pvac14::vac14::TagRFP-T::TtrpC*	This study
pccg1vac14-TagRFP-T_nat	*amp*^{R}, *ura3*, *nat*^{R}<br>*Pccg1::vac14::TagRFP-T::TtrpC*	This study
pccg1vac14-TagRFP-T_hyg	*amp*^{R}, *ura3*, *hyg*^{R}<br>*Pccg1::vac14::TagRFP-T::TtrpC*	This study
pTagRFP-T-vac14	*amp*^{R}, *nat*^{R}<br>*Pvac14::TagRFP-T::vac14::Tvac14*	This study

nat^R: nourseothricin resistant, *hyg*^{R}: hygromycin resistant, *amp*^{R}: ampicillin resistance, *kan*^{R}: kanamycin resistance, *ura3*: orotidine-5'-phosphate decarboxylase gene of *Saccharomyces cerevisiae*, *hph*: hygromycin B phosphotransferase gene, 5': represents the native promoter of the respective gene, bp: base pairs, ORF: open-reading frame, *P*: promoter, *T*: terminator, *Pgpd*: promoter of the glyceraldehyde-3-phosphate dehydrogenase gene from *A. nidulans*, *Pccg1*: promoter of the *clock-controlled gene 1* from *N. crassa*, *TtrpC*: terminator of the anthranilate synthase gene from *A. nidulans*, *egfp*: gene for green fluorescence protein enhanced green fluorescent protein (EGFP) from *Aequorea Victoria*, *TagRFP-T*: gene for red fluorescence protein TagRFP-T of *Entacmaea quadricolor*, *tdTomato*: gene for red fluorescence protein tdTomato from *Discosoma sp*.

Tab. S2 List of primers used in this study

Oligo name	Sequence (5'→3')
Vac14-ko-5f_3w	***GACTGGTCTCA AGTC*** CCTGGAGCGTACCATACGTGA
Vac14-ko-5r_3w	***CAGAGGTCTCA GCAG*** AGTCGACCTCGTCCCCATCCT
Vac14-ko-3f_3	***GTACGGTCTCG GTCA*** GTTCTTTGTAGCGCTTTTCCG
Vac14-ko-3r_3	***CTCAGGTCTCC CGTA*** TCATTGCTGGGCTCGCTTGCC
GG_KO_fw	TAGGGCGAATTGGGTACCG
GG_KO_rv	GGCCGCTCTAGAACTAGTG
GFP-f	ATGGTGAGCAAGGGCGAGGA
pRS426GFPrev	***GCGGATAACAATTTCACACAGGAAACAGC*** TCGAGTGGAGATGTGGAGTG
Vac14-tRFP-r	***TTAATCAGCTCTTCGCCCTTAGACACCAT*** TTGCTGGGCTCGCTTGCCTC
RFP-f	ATGGTGTCTAAGGGCGAAGAG
pRSccg1	***GTAACGCCAGGGTTTTCCCAGTCACGACG*** TAGAAGGAGCAGTCCATCTG
Pccg1-r	TTTGGTTGATGTGAGGGGTT
Vac14-ccg1-f	***CACTTTCACAACCCCTCACATCAACCAAA*** ATGGACGCGAACATTCAGCG
Vac14-2v5f	TAATGAGGTGCTTCTGGCAT
Vac14-2vORF5-r	AGATTTTTCTGGCGACTGGT
tC1_o	CCTGGACGACTAAACCAAAA
Vac14-2v3r	CCGTGATCTTTTCCCCCTCC
Smku70-v1-f	CATCGAGGTGAGCAAGTCAATG
ku70-ko-v3f(R)	GCGCAACTCCAGCGTGACTG
Tub2Ztf	***GTAACGCCAGGGTTTTCCCAGTCACGACG*** GCAGTCGACGCCAGATGATG
Tub2Ztr	***GCGGATAACAATTTCACACAGGAAACAGC*** GAGGAGTCGACAGCCAAGCT
Vma1P-f	***GTAACGCCAGGGTTTTCCCAGTCACGACG*** ACGGTTCTTGCAAATGGGTT
Vma1P-EGFP-r	***GTGAACAGCTCCTCGCCCTTGCTCACCAT*** GCATGCAATCTGTCTGTTCC
Vma1-EGFP-f	***TCACTCTCGGCATGGACGAGCTGTACAAG*** CAGGCGGGATTCAGACCG
Vma1-r	***GCGGATAACAATTTCACACAGGAAACAGC*** GCAGCAGCAGCAGCAGTAGC
RFP-r	CTTGTACAGCTCGTCCATGC
TtrpC_F	TCCACTTAACGTTACTGAAAT
N-vac14_P-f	***GATCTTCCGGATGGC*** AAGCAGCACGTCCAACAGTC
N-vac14_P-r	GGCTCAGAAGGGTCTCGTCGG
N-tRFP-f	***AGACCCTTCTGAGCC*** ATGGTGTCTAAGGGCGAAGAG
N-tRFP-r	CTTGTACAGCTCGTCCATGCC
N-vac14-f	***GACGAGCTGTACAAG*** ATGGACGCGAACATTCAGC
N-vac14_T-r	***ATGCCCTGCCCCTGA*** GGATTAAAACGCTGACGGGAC

Bold italics = overhangs

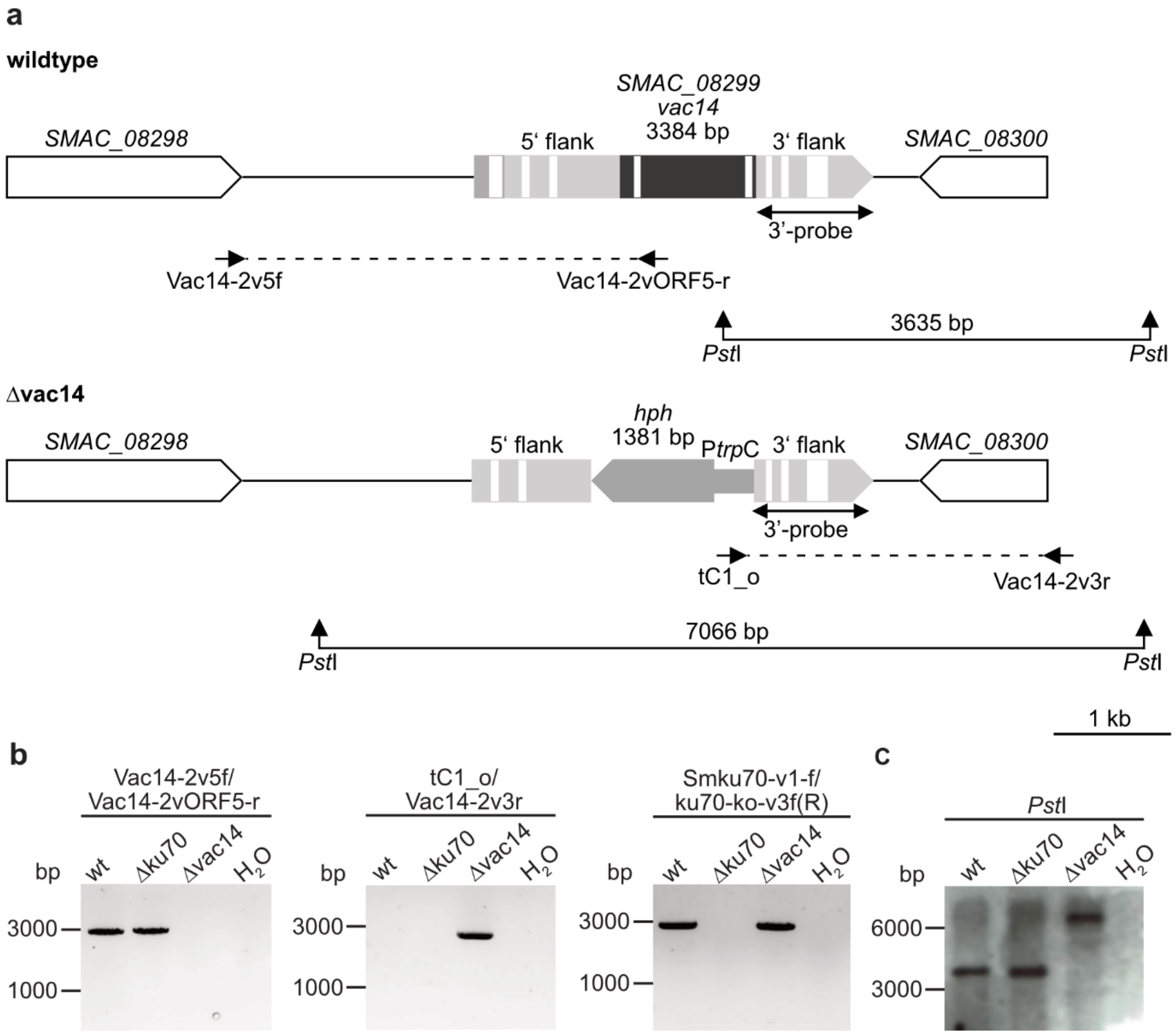

Fig. S1 Verification of partial *vac14* deletion by PCR and Southern blot analyses.

a Schematic representation of the *vac14* ORF SMAC_08299 (light grey arrow) with 8 introns (white boxes). The dark grey box indicates the region of the *vac14* ORF that is partially replaced by the *hph* cassette after Golden Gate cloning. White arrows indicate adjacent ORFs. Primer combinations, probe for Southern hybridization and hybridization regions of *Pst*I including corresponding fragment sizes are indicated. **b** PCR verification of the integration of the *hph* cassette into the *vac14* locus. Genomic DNA was isolated from the wt, Δku70 and Δvac14 strain and tested with given primer combinations. Water served as negative control. The Δku70 strain was used as a control to demonstrate the *ku70* recovery in the Δvac14 deletion strain. **c** Confirmation of the *vac14* deletion in single spore isolate 3.3 was performed by Southern blot. The isolated genomic DNA was digested with *Pst*I. Signals detected correspond to the fragment size of 3635 bp for the wt and Δku70 strain and 7066 bp for the Δvac14 deletion strain.

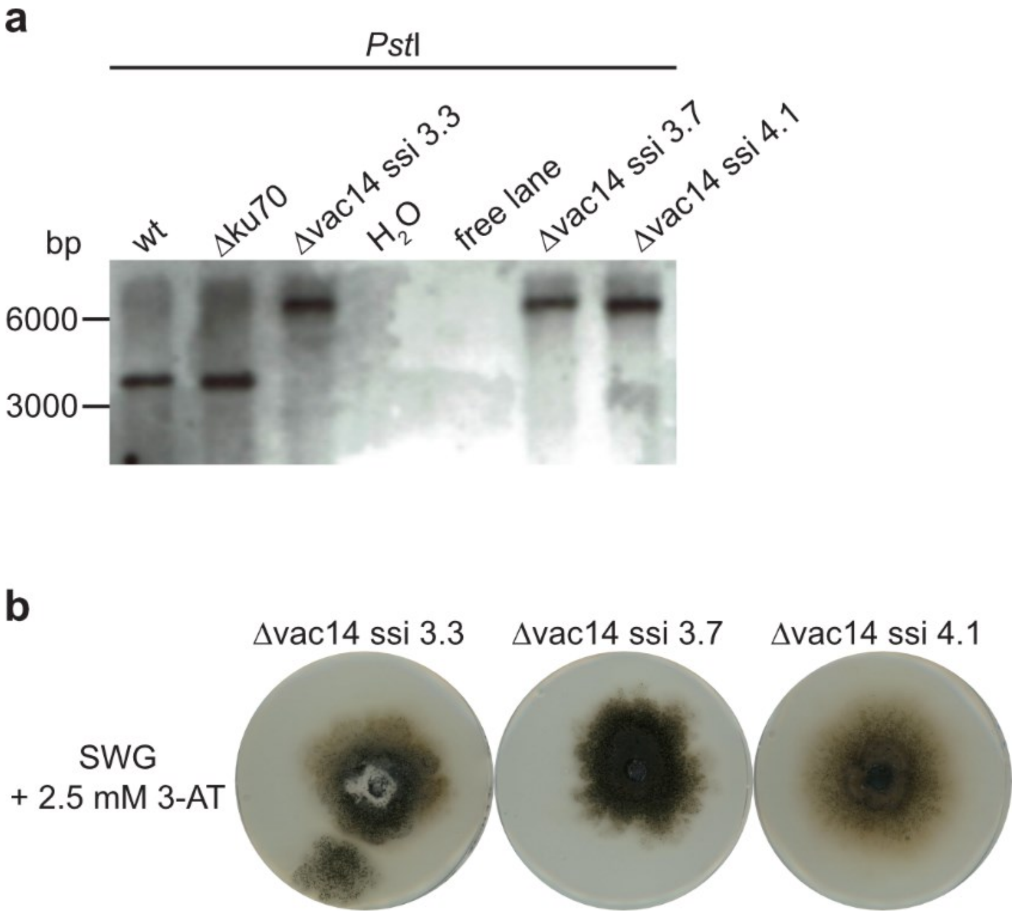

Fig. S2 Verification of two further Δvac14 single spore isolates by Southern blot analyses and growth defect of Δvac14 single spore isolates on media containing 3-AT.

a Confirmation of the *vac14* deletion in single spore isolate 3.3, 3.7 and 4.1 was performed by Southern blot. The isolated genomic DNA was digested with *Pst*I. Signals detected correspond to the fragment size of 3635 bp for the wt and Δku70 strain and 7066 bp for the Δvac14 deletion strains. **b** Growth of Δvac14 single spore isolates on media containing 3-AT.

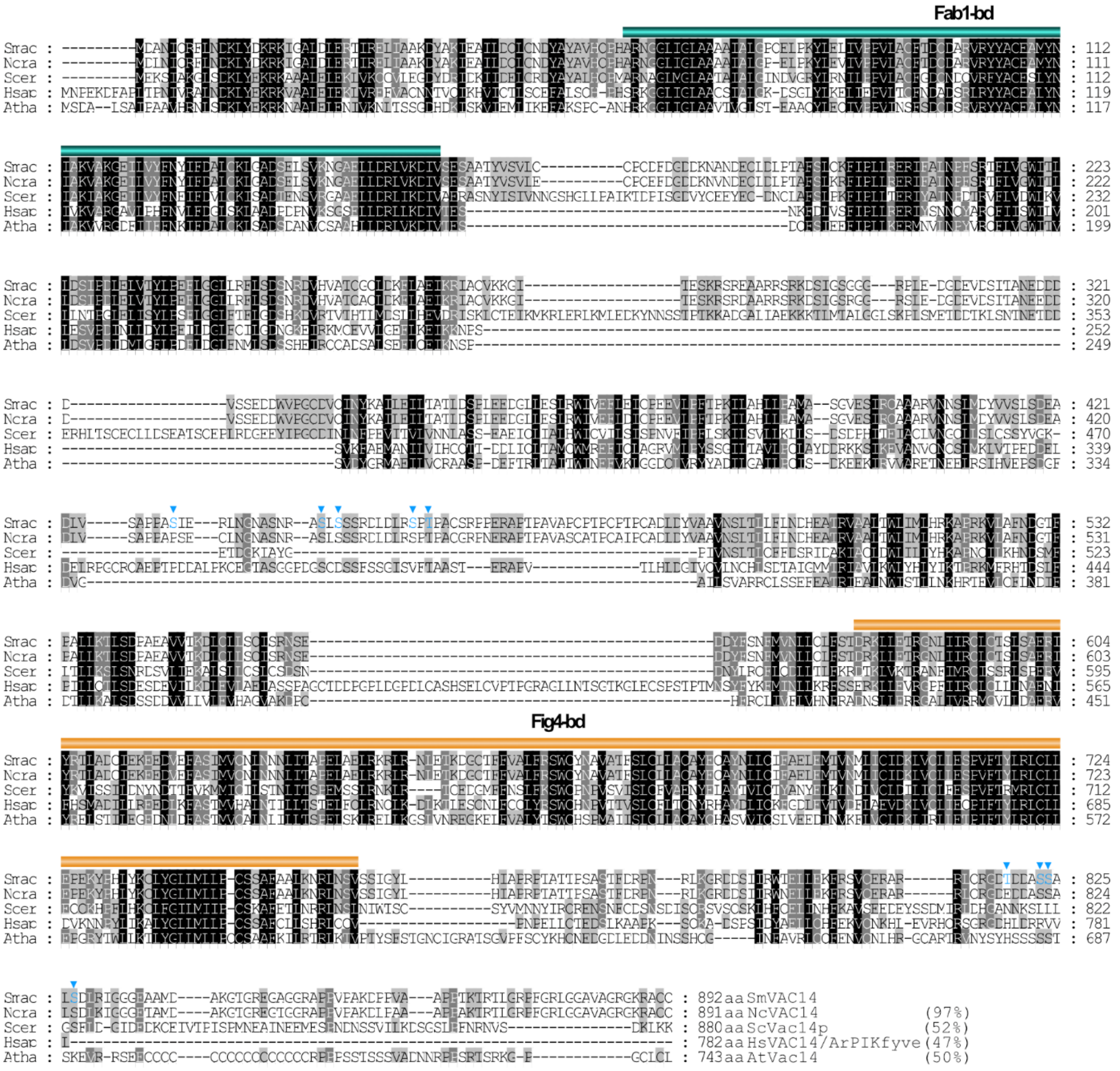

Fig. S3 Multiple sequence alignment of VAC14 homologs in different species.

Alignment of full-length VAC14 homologs. Smac, *Sordaria macrospora* (SMAC_08922) taken from the *S. macrospora*-specific peptide database Smacrospora_v03 (Blank-Landeshammer et al. 2019); Ncra, *Neurospora crassa* (XP_011395167.1); Scer, *Saccharomyces cerevisiae* (NP_013490.3); Hsap, *Homo sapiens* (NP_060522.3); and Atha, *Arabidopsis thaliana* (NP_565275.1). Sequence similarities to SmVAC14 of *S. macrospora* are indicated in percentage in brackets at the end of the protein sequence. Amino acids (aa) conserved in all proteins are shaded in black, in five of six sequences in dark grey and in four of six sequences in light grey. The N-terminal Fab1-binding domain (Fab1-bd) is shown in cyan, the C-terminal Fig4-bd in orange. Light blue triangles indicate potential phosphorylation sites after (Märker et al. 2020) and (Stein et al. 2020).

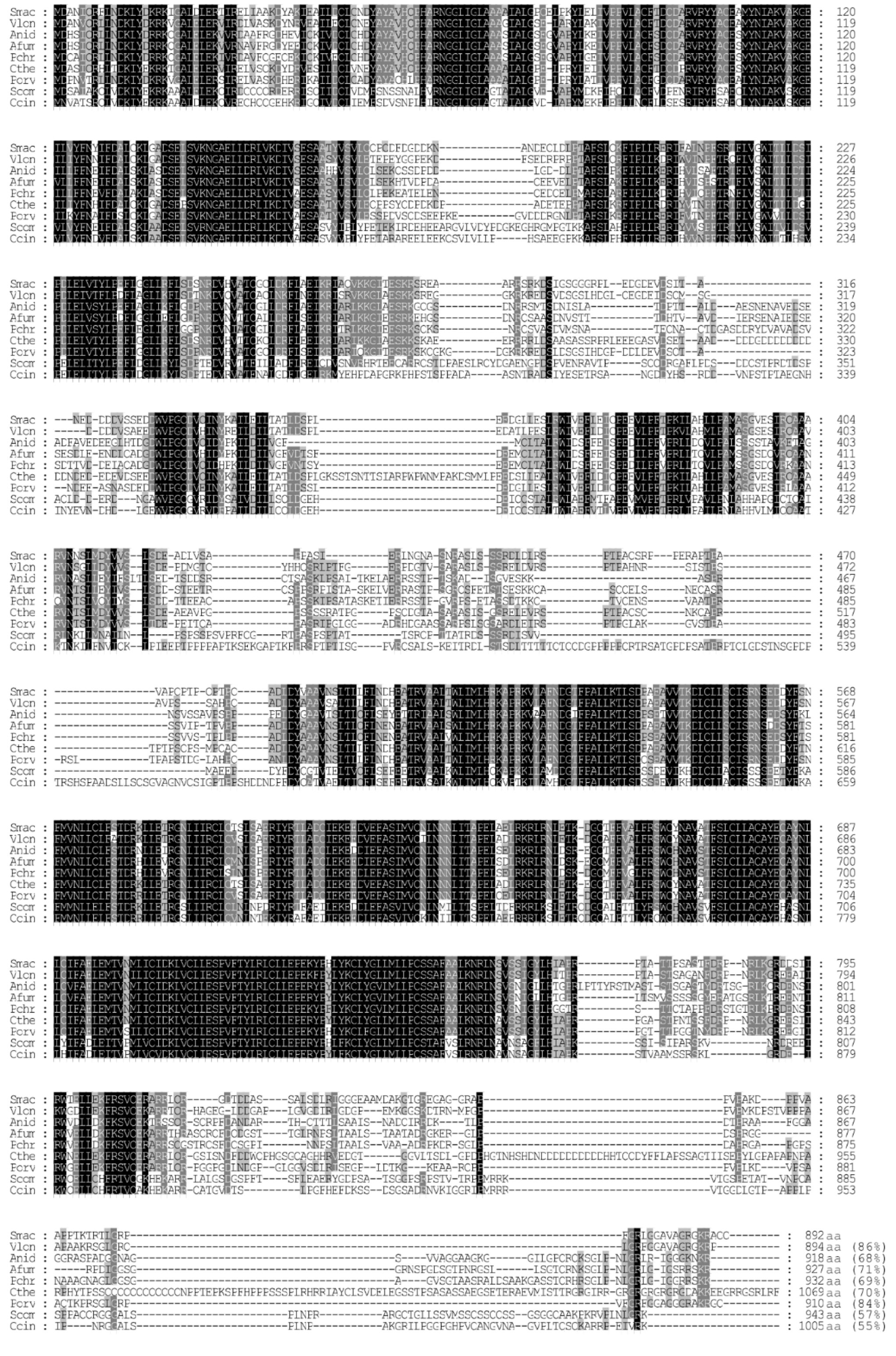

Fig. S4 Multiple sequence alignment of VAC14 proteins in fungal species.

Ascomycota: Smac, *Sordaria macrospora* (SMAC_08922) taken from the *S. macrospora*-specific peptide database Smacrospora_v03 (Blank-Landeshammer et al. 2019); Vlon, *Verticillium longisporum* (KAG7121875.1); Anid, *Aspergillus nidulans* (CBF81734.1); Afum, *Aspergillus fumigatus* (KAF4276542.1); Pchr, *Penicillium chrysogenum* (KZN86095.1); Cthe, *Chaetomium thermophilum* (XP_006695219.1) and Pory, *Pyricularia oryzae* (XP_003716347.1). Basidiomycota: Scom,

Schizophyllum commune (XP_003038141.1) and Ccin, *Coprinopsis cinerea* (KAG2013354.1). Amino acids (aa) conserved in all proteins are shaded in black, in nine or eight of ten sequences in dark grey and in seven of ten sequences in light grey. Sequence similarities to SmVAC14 of *S. macrospora* are indicated in percentage in brackets at the end of the protein sequence.

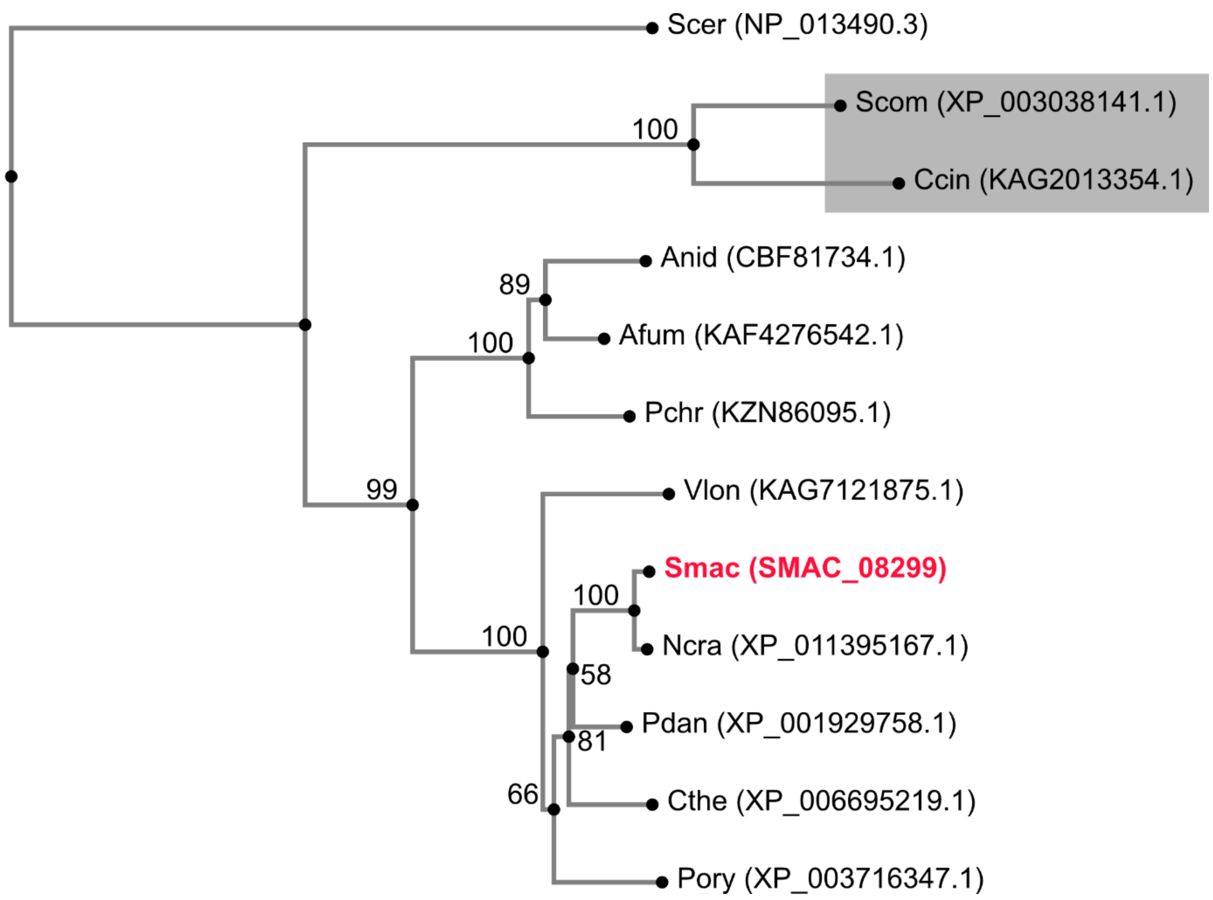

Fig. S5 Phylogenetic tree of Vac14 orthologs from fungi.

The phylogenetic tree of Vac14 was generated using the Neighbor Joining method. Orthologs were identified with BLASTP search using amino acid sequences of the *Sordaria macrospora* SmVAC14 (SMAC_08299) indicated in red. The multiple sequence alignment and phylogenetic analysis was performed with MAFFT (version 7, https://mafft.cbrc.jp/alignment/server/; accessed on 24.08.2021) (Katoh et al. 2019). Bootstrap values of 1000 replications are percentages rounded to whole numbers and are indicated at the nodes. Accession numbers are indicated. Ascomycota: Smac, *Sordaria macrospora* (SMAC_08922) from the *S. macrospora*-specific peptide database Smacrospora_v03 (Blank-Landeshammer et al. 2019); Ncra, *Neurospora crassa* (XP_011395167.1); Vlon, *Verticillium longisporum* (KAG7121875.1); Pans, *Podospora anserina* (XP_001929758.1); Anid, *Aspergillus nidulans* (CBF81734.1); Afum, *Aspergillus fumigatus* (KAF4276542.1); Pchr, *Penicillium chrysogenum* (KZN86095.1); Cthe, *Chaetomium thermophilum* (XP_006695219.1) and Pory, *Pyricularia oryzae* (XP_003716347.1). Basidiomycota: Scom, *Schizophyllum commune* (XP_003038141.1) and Ccin, *Coprinopsis cinerea* (KAG2013354.1) are shaded in grey.

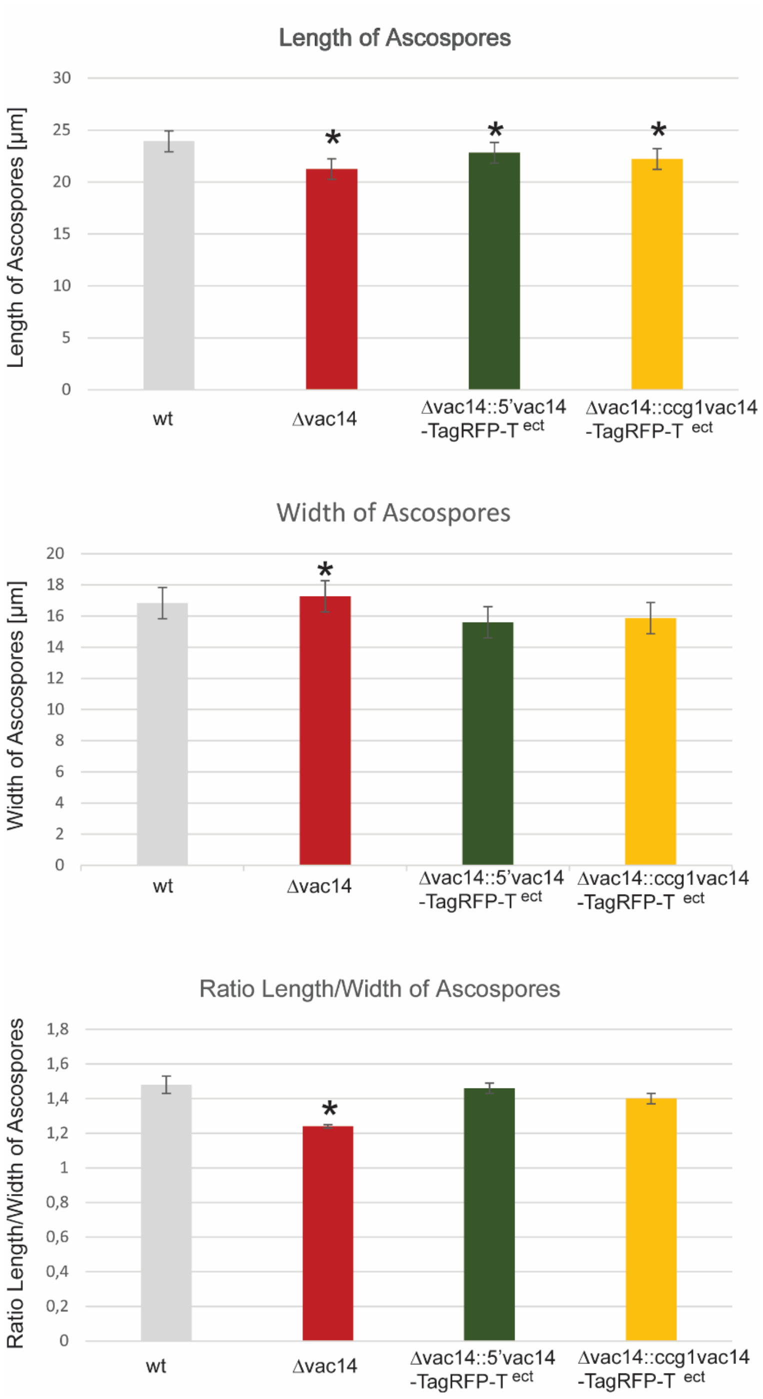

Fig. S6 Measurement of ascospores.

For measurements of ascospores, the length and width was determined for 2 complete asci (in total 16 spores) for three biological replicates of each strain (in total 48 spores for each strain). Asterisks (*) indicate a significant difference to the wt strain, according to Student's t-test ($p < 0.05$).

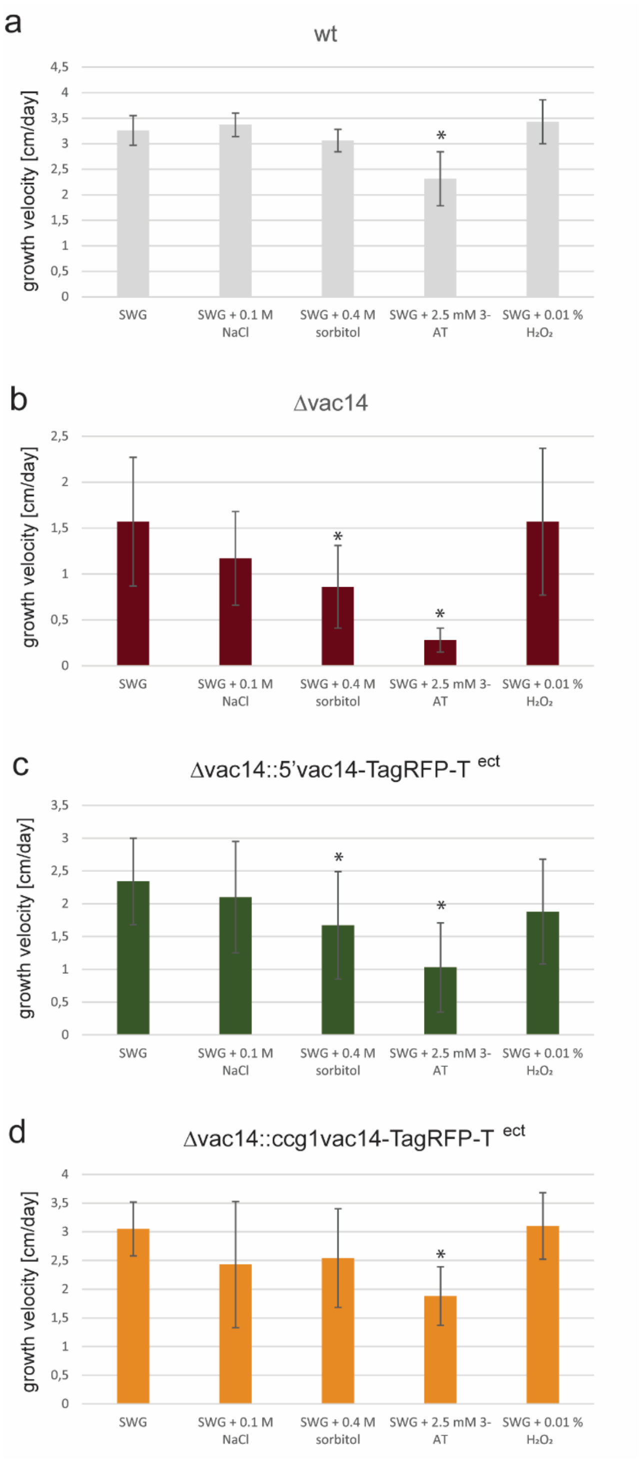

Fig. S7 Vegetative growth rate of *S. macrospora* wt, Δvac14 and the complementation strains Δvac14::5'vac14-TagRFP-T^{ect} and Δvac14::ccg1vac14-TagRFP-T^{ect} on different stress media.

For determination of growth rate per day, strains were grown in 30-cm race tubes filled with selected stress media. Three biological replicates of each strain were analyzed in three independent experiments (n = 9). Asterisks (*) indicate a significant difference to the standard growth conditions on SWG medium, according to Student's t-test ($p < 0.05$). **a** wt; **b** Δvac14, **c** Δvac14::5'vac14-TagRFP-T^{ect}; **d** Δvac14::ccg1vac14-TagRFP-T^{ect}

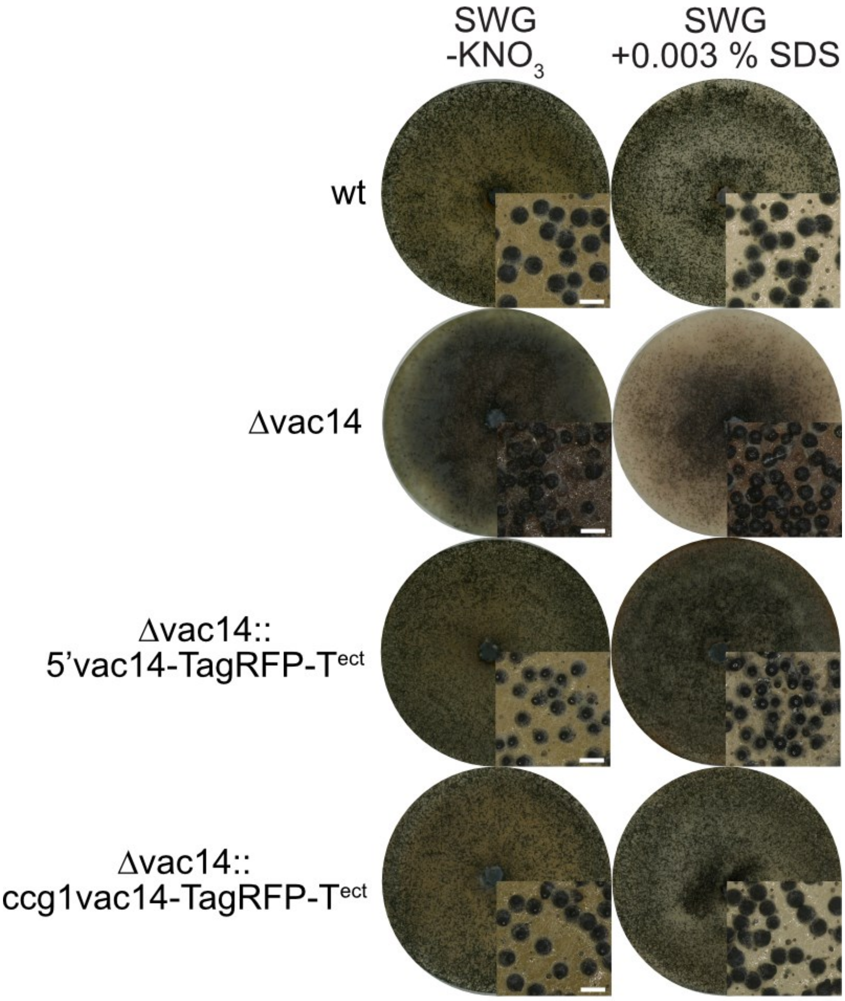

Fig. S8 Sexual developmental and growth behavior of *S. macrospora* wt, Δvac14 and the complementation strains Δvac14::5'vac14-TagRFP-T^{ect} and Δvac14::ccg1vac14-TagRFP-T^{ect} on stress media.

Strains were grown on limitation of nitrogen (-KNO_3) and under cell-wall stress conditions (+0.003 % SDS) on SWG medium. Pictures of the agar plates and enlargement of perithecia by microscopic images were taken after 10 days. Scale bar of microscopic images: 0.5 mm.

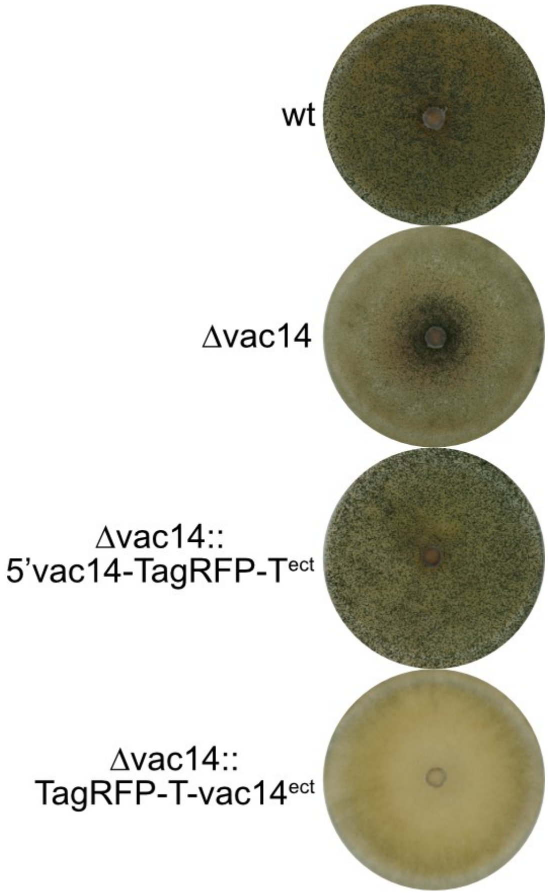

Fig. S9 Sexual developmental of *S. macrospora* wt, Δvac14 and complementation strains.

Strains were grown on SWG medium for 7 days at 27 °C under continuous light conditions. The Δvac14 mutant was complemented with SmVAC14 either C-terminally (Δvac14::5'vac14-TagRFP-T^{ect}) or N-terminally (Δvac14::TagRFP-T-$vac14^{ect}$) fused to TagRFP-T.

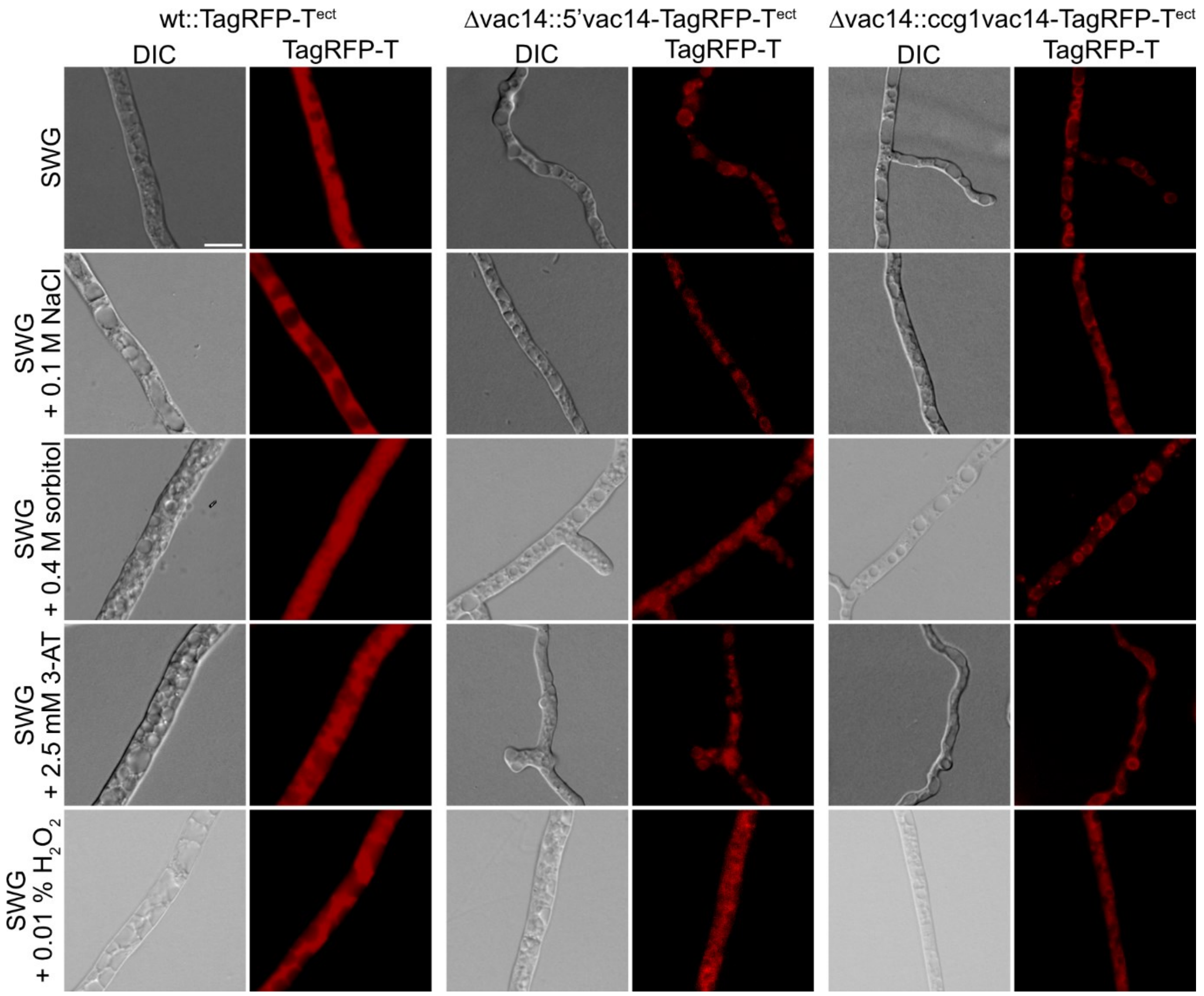

Fig. S10 Localization of native and overexpressed VAC14-TagRFP-T and free TagRFP-T in *S. macrospora* wt and Δvac14 on different stress media.

The wt::TagRFP-T^{ect} strain, expressing free TagRFP-T served as control. Strains were grown in presence of various stress conditions, such as osmotic- (0.1 M NaCl, 0.4 M sorbitol) or oxidative stress (0.01 % H_2O_2) or under amino-acid starvation (25 mM 3-AT) by adding the components to SWG + 1.5 % agarose medium. Scale bars = 10 µm, DIC: differential interference contrast.

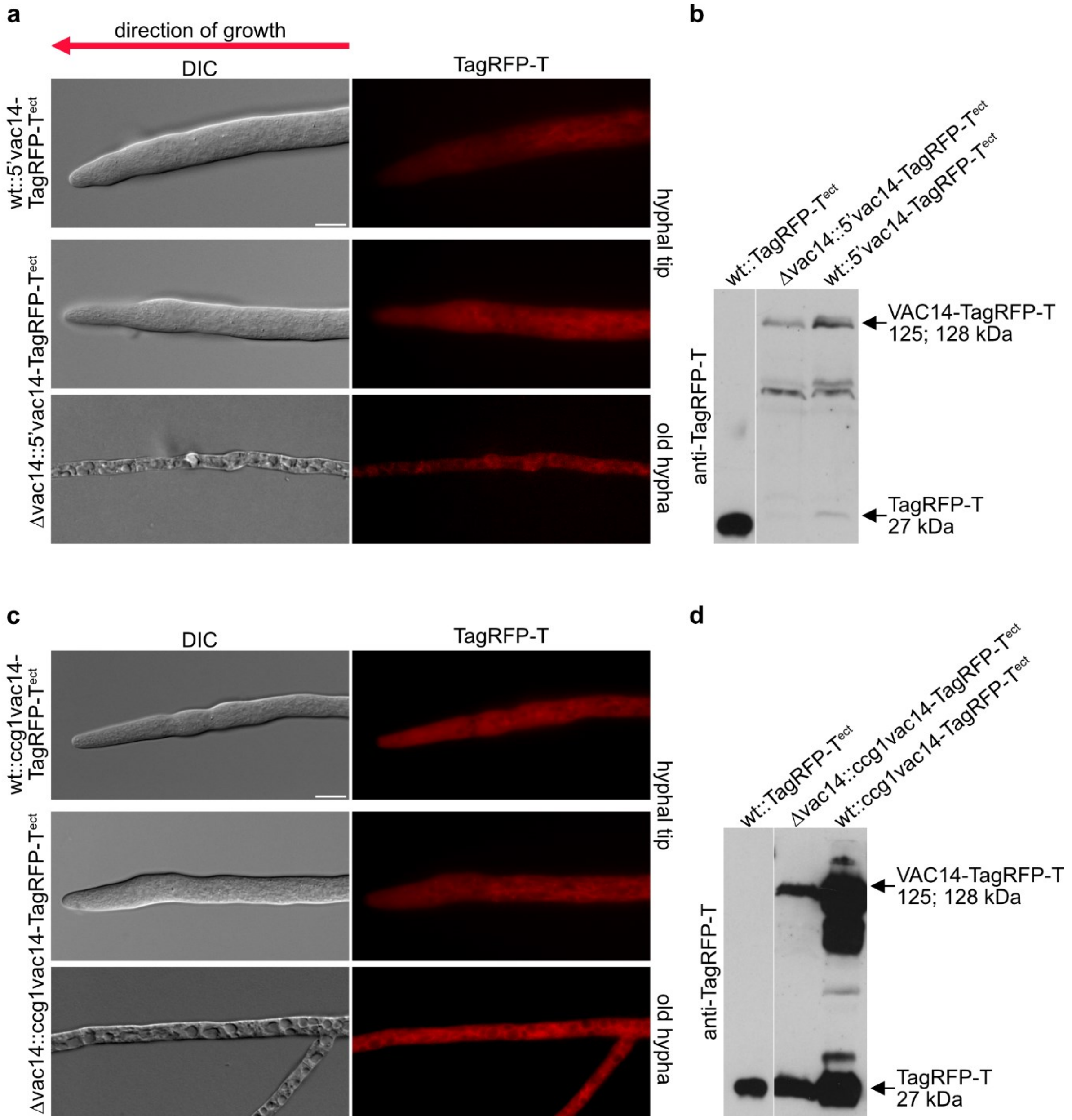

Fig. S11 Localization of native and overexpressed VAC14-TagRFP-T in *S. macrospora* wt and Δvac14 strains

For Fluorescence microscopy, strains were grown on BMM-slides for 24 h or on solid SWG + 1.5 % agarose medium for 24 h at 27 °C under continuous light to visualize young and old hyphae, respectively. **a** Localization of 5'VAC14-TagRFP-T under the control of the endogenous promoter in *S. macrospora* wt and Δvac14. **b** Corresponding Western blot analysis. **c** Localization of overexpressed ccg1VAC14-TagRFP-T, with *vac14* under the control of the overexpression promoter ccg1 of *N. crassa*, in wt and Δvac14 strains. **d** Corresponding Western blot analysis. The wt::TagRFP-T^{ect} strain expressing free TagRFP-T served as control in Western blot experiments. Protein sizes are indicated. Degradation products of the VAC14-fusion protein are visible in **b** and **d**. A putative dimerization band is visible in **d**. Scale bars = 10 µm, DIC: differential interference contrast.

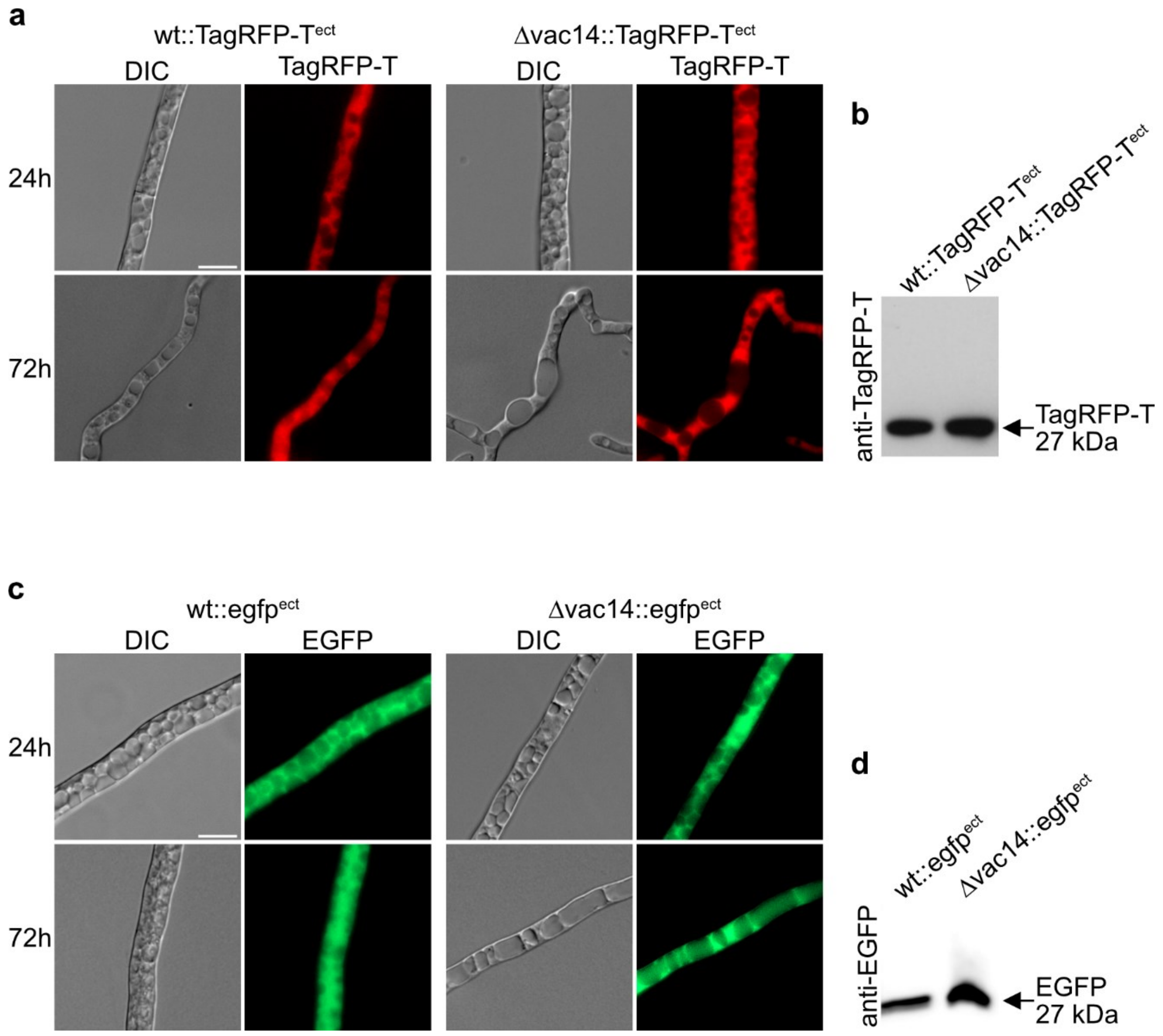

Fig. S12 Localization of free TagRFP-T and EGFP in *S. macrospora* wt and Δvac14

Strains were grown on BMM-slides for 24 h or on SWG + 1.5 % agarose for 72 h at 27 °C under continuous light for fluorescence microscopic analysis. **a** *S. macrospora* wt and Δvac14 carrying pTagRFP-T with *TagRFP-T* under the control of the *N. crassa ccg1* promoter (Werner et al. 2021) and corresponding Western blot analysis (**b**) are shown. **c** *S. macrospora* wt carrying p1783-1 (Pöggeler et al. 2003) and Δvac14 carrying pDS23 (Teichert et al. 2012) with *egfp* under the control of the *A. nidulans gpd* promoter, respectively. Corresponding Western blot experiment is shown in **d**. Protein sizes are indicated. Scale bars = 10 µm, DIC: differential interference contrast.

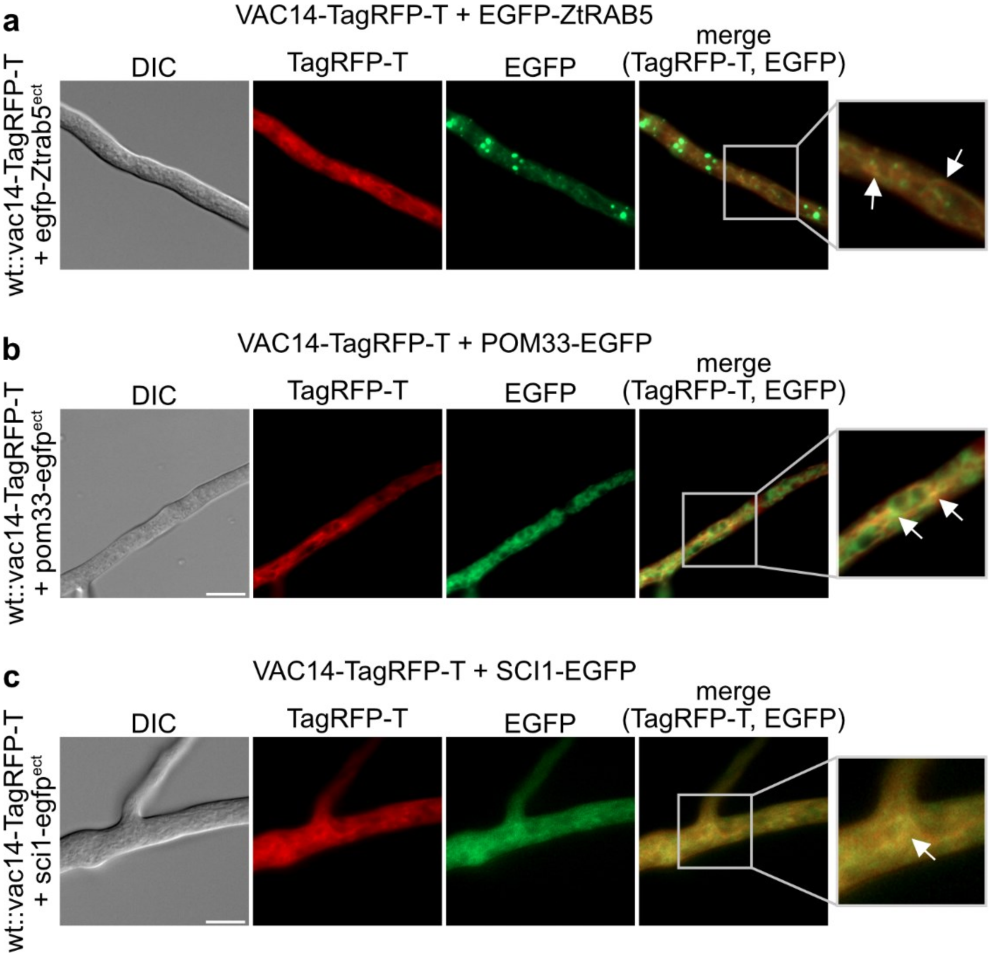

Fig. S13 Co-localization of VAC14 and early endosomal, ER and STRIPAK marker proteins in apical hyphal compartments of the growth front using different fluorescence tags.

S. macrospora wt was either co-transformed or strains expressing fluorescent proteins were crossed. Fluorescence microscopy was performed to visualize co-localization of the fusion proteins. **a** *S. macrospora* wt expressing VAC14-TagRFP-T together with the fluorescence-tagged *Z. tritici* early endosomal marker EGFP-ZtRAB5**.** White arrows indicate putative co-localization. **b** *S. macrospora* wt strains expressing either VAC14-TagRFP-T or the ER-marker protein POM33-EGFP were crossed and putative co-localization of both fusion proteins is indicated by white arrows. **c** *S. macrospora* wt was co-transformed to express VAC14-TagRFP-T and the fluorescence-labeled SmSTRIPAK-component SCI1-EGFP. A white arrow indicates a putative co-localization of both fusion constructs. Scale bars = 10 µm, DIC: differential interference contrast. Detailed two-fold enlargements of the merge pictures are indicated by a frame and shown at the right margin.

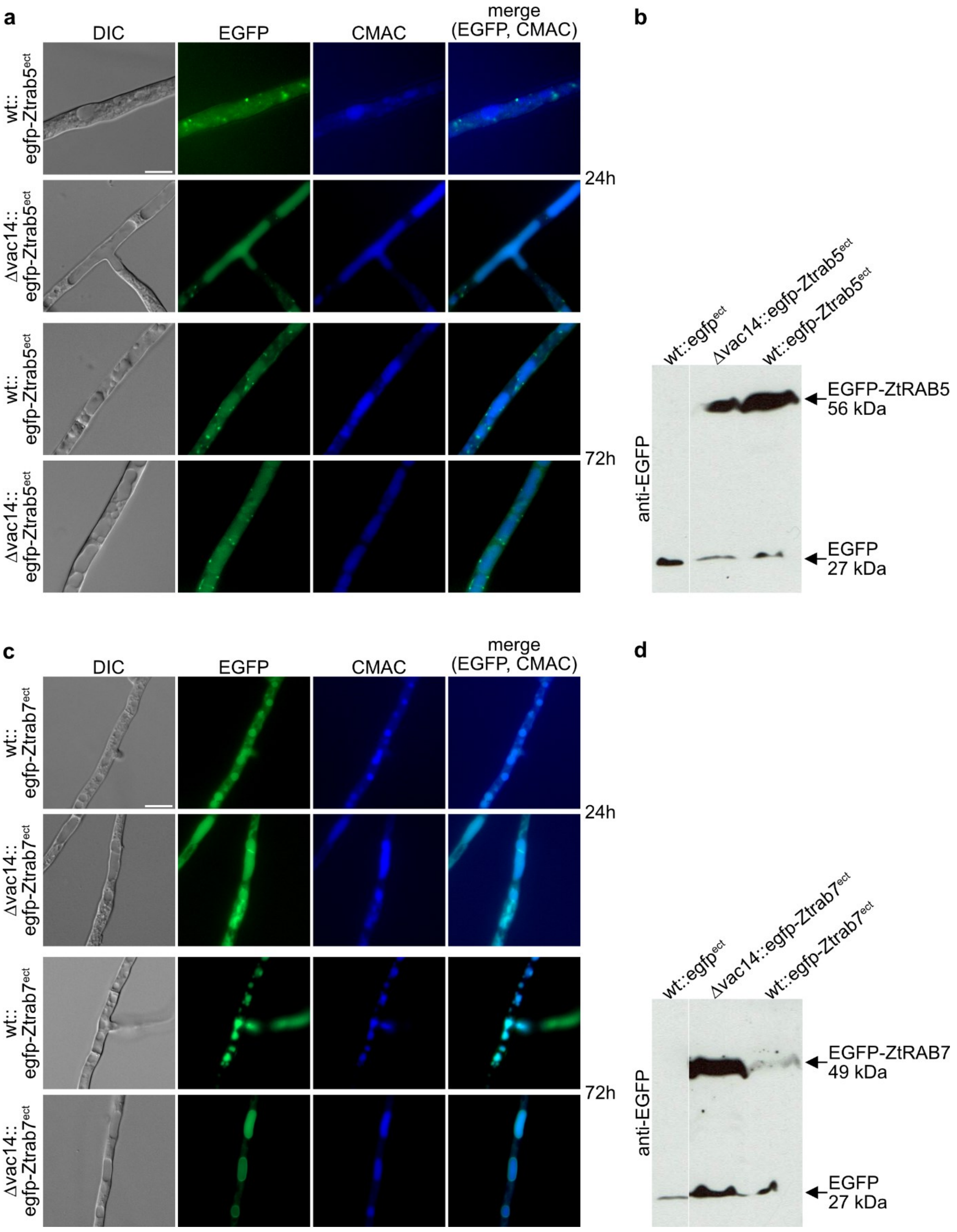

Fig. S14 Localization of the early- and late-endosomal markers ZtRAB5 and ZtRAB7 in older sub-apical compartments of *S. macrospora* wt and Δvac14 strains.

S. macrospora wt and Δvac14 strains expressing the fluorescence-tagged *Z. tritici* early- and late-endosomal markers EGFP-ZtRAB5 (**a**) and EGFP-ZtRAB7 (**c**) with corresponding Western blot analysis (**b**) and (**d**), respectively. Strains were grown on

BMM-slides for 24 h (complete medium, healthy young mycelium) and on SWG-slides for 72 h (defined medium with less nutrients, aged mycelium) at 27 °C under continuous light. Vacuoles of the hyphae were stained with CMAC (1:400 of 10 mM stock solution) and incubated for 30 min at 37 °C. Scale bars = 10 µm, DIC: differential interference contrast.

Video S1 and S2 Localization of nuclei in growing hyphal tips

Heterologous expression of pRH2B_nat (Reschka et al. 2018) in *S. macrospora* wt (S1) and Δvac14 (S2) hyphae. Recording of hyphae from growing strains on BMM + agarose medium after incubation for 24 h at 27 °C, recording interval = 5 s, size bar = 20 μm.

References

Klix V, Nowrousian M, Ringelberg C, Loros JJ, Dunlap JC, Pöggeler S (2010) Functional characterization of MAT1-1-specific mating-type genes in the homothallic ascomycete *Sordaria macrospora* provides new insights into essential and nonessential sexual regulators. Eukaryot Cell 9:894-905. 10.1128/EC.00019-10

Bloemendal S, Bernhards Y, Bartho K, Dettmann A, Voigt O, Teichert I, Seiler S, Wolters DA, Pöggeler S, Kück U (2012) A homologue of the human STRIPAK complex controls sexual development in fungi. Mol Microbiol 84:310-323. 10.1111/j.1365-2958.2012.08024.x

Pöggeler S, Masloff S, Hoff B, Mayrhofer S, Kück U (2003) Versatile EGFP reporter plasmids for cellular localization of recombinant gene products in filamentous fungi. Curr Genet 43:54-61. 10.1007/s00294-003-0370-y

Teichert I, Wolff G, Kück U, Nowrousian M (2012) Combining laser microdissection and RNA-seq to chart the transcriptional landscape of fungal development. BMC Genomics 13:511. 10.1186/1471-2164-13-511

Werner A, Otte K, Stahlhut G, Hanke LM, Pöggeler S (2021) The Glyoxysomal Protease LON2 Is Involved in Fruiting-Body Development, Ascosporogenesis and Stress Resistance in *Sordaria macrospora*. J Fungi (Basel) 7. 10.3390/jof7020082

Dahlmann TA, Terfehr D, Becker K, Teichert I (2021) Golden Gate vectors for efficient gene fusion and gene deletion in diverse filamentous fungi. Curr Genet 67:317-330. 10.1007/s00294-020-01143-2

Reschka EJ, Nordzieke S, Valerius O, Braus GH, Pöggeler S (2018) A novel STRIPAK complex component mediates hyphal fusion and fruiting-body development in filamentous fungi. Mol Microbiol 110:513-532. 10.1111/mmi.14106

Kilaru S, Schuster M, Latz M, Guo M, Steinberg G (2015) Fluorescent markers of the endocytic pathway in *Zymoseptoria tritici*. Fungal Genet Biol 79:150-157. 10.1016/j.fgb.2015.03.019

Werner A, Herzog B, Voigt O, Valerius O, Braus GH, Pöggeler S (2019) NBR1 is involved in selective pexophagy in filamentous ascomycetes and can be functionally replaced by a tagged version of its human homolog. Autophagy 15:78-97. 10.1080/15548627.2018.1507440

Voigt O, Pöggeler S (2013) Autophagy genes Smatg8 and Smatg4 are required for fruiting-body development, vegetative growth and ascospore germination in the filamentous ascomycete *Sordaria macrospora*. Autophagy 9:33-49. 10.4161/auto.22398

Blank-Landeshammer B, Teichert I, Marker R, Nowrousian M, Kück U, Sickmann A (2019) Combination of Proteogenomics with Peptide De Novo Sequencing Identifies New Genes and Hidden Posttranscriptional Modifications. mBio 10. 10.1128/mBio.02367-19

Märker R, Blank-Landeshammer B, Beier-Rosberger A, Sickmann A, Kück U (2020) Phosphoproteomic analysis of STRIPAK mutants identifies a conserved serine phosphorylation site in PAK kinase CLA4 to be important in fungal sexual development and polarized growth. Mol Microbiol 113:1053-1069. 10.1111/mmi.14475

Stein V, Blank-Landeshammer B, Muntjes K, Märker R, Teichert I, Feldbrugge M, Sickmann A, Kück U (2020) The STRIPAK signaling complex regulates dephosphorylation of GUL1, an RNA-binding protein that shuttles on endosomes. PLoS Genet 16:e1008819. 10.1371/journal.pgen.1008819

Katoh K, Rozewicki J, Yamada KD (2019) MAFFT online service: multiple sequence alignment, interactive sequence choice and visualization. Brief Bioinform 20:1160-1166. 10.1093/bib/bbx108
